# Self-reported prevalence of tuberculosis and selected associated vulnerability factors among rural populations in six districts of India: A community-based cross-sectional study

**DOI:** 10.1371/journal.pone.0360758

**Published:** 2026-10-16

**Authors:** P K Anand, Manjula Singh, Purbita Banerjee, Seema Sahay, Sampada Bangar, Braj Raj Ghosh, Manjeet Singh Chalga, Anurag Pappu, VNR Das, Sarika Gupta, Amber Kumar, Dhruvendra Pandey

**Affiliations:** 1 ICMR – National Institute of Health Research, Jodhpur, Rajasthan, India; 2 Indian Council of Medical Research (ICMR), Ministry of Health and Family Welfare, India; 3 Indian Institute of Public Health, Gandhinagar, Gujarat, India; 4 ICMR – National AIDS Research Institute, Pune, India; 5 ICMR – Rajendra Memorial Research Institute on Medical Sciences, Patna, India; 6 King George Medical University, Lucknow, India; 7 AIIMS, Bhopal, India; 8 Government Medical College, Ratlam, India; National Institute of Tuberculosis and Respiratory Diseases, INDIA

## Abstract

**Background:**

Tuberculosis is a major public health challenge in India, contributing to one-fourth of global burden of TB disease. Comorbidities in uninfected individuals make them vulnerable to TB disease. This study estimates the self-reported prevalence of tuberculosis and selected vulnerability factors (HIV, current smoking, silicosis, occupational silica-dust exposure, diabetes mellitus and persistent cough >2 weeks) among the rural demography of six districts in India, with the aim of informing targeted active case finding under the National Tuberculosis Elimination Programme (NTEP).

**Methods:**

The study conducted was a community-based cross-sectional survey carried out across six districts of India. Two blocks from each district was selected randomly followed by randomly selected villages under each block. A universal sampling technique was followed for including all the households of the randomly selected villages. The data were collected through a pre-tested semi-structured questionnaire schedule via face-to-face interviews at all the eligible households of the study sites. Descriptive analysis was performed, and the results were obtained in the form of frequencies and percentages.

**Results:**

The ultimate data consisted of 82,636 individuals, with males at 52% and females at 47%. 77% of the study population were 15 years of age or older. This study analyzes a large dataset. The TB prevalence was around 150 per lakh of the population, while the prevalence of HIV-reactive individuals, current smokers, silicosis, occupational exposure to silica dust, diabetes mellitus and cough of greater than two weeks is found in 0.9%, 5%, 1.2%, 1.1%, 1.4% and 4% respectively.

**Discussion and conclusion:**

The study highlights TB status and selected TB-vulnerable groups. The study has a large sample size, and it follows multipronged approach to study selected vulnerable population groups. This data may improve targeted active case finding among the high-risk groups as outlined by NTEP and provide better TB control strategy outcome at high TB burden settings.

## Introduction

TB is the 10^th^ leading cause of death worldwide and 2^nd^ leading cause of death from a single infectious agent worldwide after COVID-19 [1]. According to the Global TB Report 2025, India accounts for 25% of the Global TB Burden which is the highest in the world [1]. Tuberculosis is directly linked with the social determinants of health. As stated by the India TB Report 2024, TB is highly influenced by socio-economic development as well as health risk factors like HIV status, smoking habits, silicosis, occupational exposure to silica dust, diabetes mellitus and symptoms like persistent cough of more than 2 weeks. In order to better manage the TB epidemic, it is essential to focus on the underlying vulnerabilities among the demographic that is driving the disease transmission.

WHO identifies key vulnerable populations as those with increased risk of TB exposure due to their living conditions, such as prisoners and migrants, individuals with weakened immune systems, and those with chronic illnesses. They face barriers to accessing healthcare services and may have poor health-seeking behaviour, which makes early identification and active case finding essential to ensure timely and effective TB care [3].

Tuberculosis is known to affect people with certain comorbidities disproportionately. Individuals who are suffering from Diabetes, People living with HIV (PLHIV), individuals with silicosis, construction site or mine workers having occupational exposure to silica dust as well as chronic smokers are included in this group [2]. The comorbidities may impact TB infection individually or overlap one another to create a much more complicated scenario. Like Diabetes is one of the risk factors of TB, known to downregulate immunity that put diabetics at a three-fold increased risk of developing active TB in comparison to non-diabetics [4]. Moreover Diabetics show complicated TB treatment outcomes as per the India TB Report 2024. The mindful focus on diagnosing, detecting and treating DM may have a beneficial impact on the control of the TB epidemic [4]. HIV another risk factor causes immunosuppression leading to twenty times at higher risk of developing TB in comparison to general population as per the India TB Report 2024. HIV is known to be responsible for 1,50,000 deaths due to TB worlwide [1]. Literature also states that silicosis and silica dust exposure is associated with increased risk of tuberculosis [5]. Then again Smoking has been established as an independent risk factor, with suppressive effects of smoke inhalation on the alveolar macrophage function as well as antibiotic resistance [2].

In India, TB is intertwined with socio-economic vulnerabilities and comorbidities, including diabetes, silicosis, occupational exposure to silica dust, smoking, and cough persisting for more than 2 weeks among other factors, which disproportionately affect marginalized and rural populations due to limited healthcare access, stigma, and delayed diagnosis. The incidence of DM is reported to be 9.6% and expected to rise to 10.9% by 2045 [6]. While a study showed 12.3% of the sandstone mine workers had radiograph indicating silicosis [7]. Though the exact proportion of the population employed by the industry of stone-mines, stone quarry or the construction sites is not known. Data on smoking showed 15.6% prevalence in India with bidi smoking being the commonest form of smoking specifically in rural areas [8]. Cough on the other hand is the cornerstone of TB diagnosis, persistent cough of over 2 weeks have higher sensitivity and lower specificity as per a TB diagnostic tool [9] while if unnoticed results in higher disease transmission rate [10]. These drivers elevate infection risk, worsen outcomes, and fuel drug resistance, challenging the National TB Elimination Programme’s aim to eliminate TB [5]. Thus ground level research is required to counter these social and high risk factors that drive the Indian TB epidemic. Moreover a study also stresses upon enhanced focus on research in India to better understand the conditions which are interlinked in order to launch effective prevention and management strategies [11]. Thus estimating the prevalence of these populations in a specific geographic backdrop can help us map TB propensity of a particular site and take specific measures in advance with respect to the TB preventive measures or bridge the gaps present in current NTEP implementation.

This study was undertaken to estimate the prevalence of TB vulnerable population in Northern India.

Objectives:

- To estimate the self-reported prevalence of tuberculosis and of selected factors that increase vulnerability to TB (HIV infection, current smoking, silicosis, occupational exposure to silica dust, diabetes mellitus and cough lasting more than two weeks) in the rural population of the six study districts.
- To describe the socio-demographic characteristics (age, gender, occupation and community group) of the study population.
- To examine the distribution of the selected vulnerability factors across socio-demographic strata.
- To produce grassroots-level evidence for strengthening targeted TB prevention and screening strategies among high-risk groups.

## Methodology

### Study design and setting

The study was a community-based cross-sectional study, conducted in six districts of India namely: Bhopal (Madhya Pradesh), Jaipur (Rajasthan), Lucknow (Uttar Pradesh), Patna (Bihar), Pune (Maharashtra) and Ratlam (Madhya Pradesh); selected on the basis of location of the collaborating centres. The study was conducted under the academic and technical supervision of Indian Council of Medical Research- National Institute of Implementation Research (ICMR-NIIRNCD), Jodhpur, Rajasthan.

### Sampling methodology

In the first stage, six districts included in the study were purposively selected as they were the established operational areas of the collaborating investigators. This provided feasibility for multi-centric fieldwork. These districts were known to have higher TB Burden and delays in care-seeking. In the second stage within each district, two blocks were selected randomly. From each selected block, list of villages with active Nehru Yuva Kendra Sangathan (NYKS) youth clubs were obtained from NYKS office. From this list of villages with NYKS youth clubs, 5 villages per block were select. This random selection of villages was done in accordance with the simple random sampling as done under the NYKS study [22]. In the fourth and final stage universal sampling was done of all the households and family members under the selected villages of the district.

### Study population

The study population included all permanent residents residing in the selected study areas/sites belonging to all age groups and genders.

Eligibility criteria of the respondent:

- All the families who were residing in the study sites were selected on household basis.
- One member from each family was selected as the household respondent for purpose of collecting information.

### Sample size and sampling technique

The final study population comprised approximately 82,636 individuals. Universal sampling was employed, wherein all eligible households in the six selected districts were surveyed to achieve complete enumeration and minimize selection bias. Households found locked were revisited twice at different time points.

### Study variables

The primary outcome was tuberculosis vulnerability, defined as reported by the people of present TB status as well as having diabetes mellitus, HIV infection, silicosis, occupational silica exposure and individuals having smoking habit. All the variables were categorised as per the operational definition chosen in the study (elaborated in Table 4). Another aspect emphasized in the study is the TB prevalence, which is calculated by adding the new and old cases of TB. New cases were represented by the “Currently receiving ATT” while the old cases were represented by “Completed ATT in the past 2 years”. Adding both of these values give us TB Prevalence. Independent variables included age group, sex, occupation, and community group.

### Data collection method and tool

Data were obtained via household-based face-to-face interviews using a pretested semi-structured questionnaire administered by trained investigators. A designated adult respondent provided information for all household members in local dialect. The tool, developed from review of relevant literature and national programme guidelines, assessed socio-demographic characteristics, diabetes mellitus, HIV infection, and silicosis or occupational silica exposure and opinion of the investigators of the study and subject experts.

### Data analysis

The data was cleaned and the study sites were geographically represented. The categorical data was expressed as percentages and frequencies.

### Ethical considerations

The study followed the ethical guidelines as prescribed by the Indian Council of Medical Research (ICMR) guidelines 2025. The Institute Ethics Committee of ICMR-NIIRNCD, Jodhpur granted the ethics approval after going over the research protocol (File No. IEC-ICMR-NIIRNCD/2021/25/3). This study was funded by the Indian Council of Medical Research (ICMR) vide File No. 5/8/5/44/ITRC/Implementation/2021/ECD-I. The funders had no role in study design, data collection and analysis, decision to publish, or preparation of the manuscript. Informed consent was obtained from all the participants.

## Results

The initial dataset comprised 84,004. Data cleaning involved removal of blank entries (n = 576), records with cough for >2 weeks (n = 112), missing district information (n = 33), and records with unverifiable respondent identification (n = 647). No missing values were observed for block or village variables. The final analytical dataset consisted of 82,636 records and was used for statistical analysis.

Table 1 illustrates the geographical distribution of the study participants in the respective study sites with the distribution of individuals among these places. The study presents information of status of TB and known vulnerable factors among 82636 individuals residing in 12736 households in 6 districts of 5 states in India. This information was collected from 12736 adult respondents found available at these households to represent their family.Table 2 presents the socio-demographic characteristics of the study population. Of the 82,636 participants, 52% were male (n = 43,134), 47% female (n = 39,478), and 1% identified as third gender (n = 24). The majority were aged ≥15 years (77%, n = 63,445), while 7% (n = 6,058) were <6 years and 16% (n = 13,133) were aged 6–15 years.. Largest occupation group with 60% share was “Not in labour force” represented by women, children and dependent senior family members. ‘Agriculture & Allied’ category of occupation was the largest group with 14% share. Among the community groups studied ‘Other Backward Classes’ scored highest rank with 44% share of study population. This group was followed by ‘Scheduled Caste’ community group with 30% share.

**Table 1:** Geographical distribution of the study participants (n = 82636).

District name	Household No. (%)	Individuals covered No. (%)
Bhopal	3187 (25%)	19372 (23.4%)
Jaipur	2169 (17%)	13122 (15.9%)
Lucknow	3868 (30%)	20222 (24.5%)
Patna	458 (3.6%)	3033 (3.7%)
Pune	1087 (8.5%)	12537 (15.2%)
Ratlam	1967 (15.4%)	14350 (17.4%)
**Total**	**12736 (100%)**	**82636 (100%)**

**Table 2:** Characteristics of the study participants (n = 82636).

Characteristics	Categories	No. (%)
**Gender**	Male	43134 (52%)
	Female	39478 (47%)
	Third Gender	24 (<1%)
**Age group**	0 to 5 years of age	6058 (7%)
	6 to 15 years of age	13133 (16%)
	Greater than 15 years of age	63445 (77%)
**Occupation**	Agriculture & Allied	11807 (14%)
	Unskilled/Manual Workers	5,764 (7%)
	Skilled/Semi-skilled Workers	6,375 (8%)
	Professional workers & Business Owners	4,186 (5%)
	Not in Labour Force	54,504 (66%)
**Community Group**	Scheduled Caste	25200 (30%)
	Scheduled Tribe	11493 (14%)
	Other Backward Classes	36073 (44%)
	General	9870 (12%)
	**Total**	**82636 (100%)**

Table 3 presents key clinical, behavioural and occupational characteristics of the study population (N = 82,636). Prior tuberculosis was reported by 0.29%of the population, and 0.08% reported recent completion of anti-tubercular treatment and ongoing treatment was found in 0.07% of the study sample. TB Prevalence is calculated from the table by adding the values under “Currently receiving ATT” and “Completed ATT within the last 2 years” which gave a total of 0.15%. As TB statuses could not be confirmed through microbiological laboratory testing, it could not be effectively classified into active TB cases and other criteria. So, the above-mentioned two categories were defined according to the self-reporting nature of the study. “Currently receiving ATT” signified active cases as they were receiving therapy, while “Completed ATT within the last 2 years” signified individuals who completed therapy recently but have a chance of relapse. The majority of relapse cases of TB occur in the first two years of treatment completion. HIV reactivity was observed in 0.9% of individuals. The majority were never smokers (94%). Self-reported silicosis (1.2%) and occupational silica exposure (<2%) were also noted among the population. Diabetes was reported by 1.4% and cough was present in 4% of participants.

**Table 3:** Distribution of Tuberculosis and other vulnerable groups among the participants (n = 82636).

TB and selected vulnerable groups.	Sub-groups	n (%)
TB status	Currently receiving ATT	60(0.07%)
	Completed ATT within last 2 years	68 (0.08%)
	Old TB	236 (0.29%)
	No TB	82272 (99.56%)
HIV	Reactive	717 (0.87%)
	Non-Reactive	81919 (99.13%)
Diabetes	Yes	1144 (1.38)
	Don’t Know	1694 (2.05)
	No	79798 (96.57)
Silicosis	Yes	990 (1.20%)
	Don’t Know	1116 (1.35%)
	No	80530 (97.45%)
Silica Exposure	Exposed to mine work	312 (0.38%)
	Exposed in building construction work	598 (0.72%)
	No Exposure	81726 (98.90%)
Smokers	Current Smoker	3888 (4.70%)
	Past Smoker	841 (1.02%)
	Never a Smoker	77907 (94.28%)
Cough >2 weeks	Cough Present	3414 (4.13%)
	No Cough	79222 (95.87%)
	**TOTAL**	**82636 (100%)**

Table 4 tabulates all the operational definitions that has been considered in the study questionnaire for assessing the health status of the individuals through self reporting. While Table 5 tabulates the prevalence, percentages and 95% CI of all the diseases and risk factors considered in the study and give the raw values with approximation. We will notice that adjustment for clustering or sampling design was not done in the study. This is because initially the districts were chosen as per the presence of collaborator centres, followed by selection of two blocks by simple random sampling. Villages under each block were again chosen by simple random sampling. Finally all the households and every member of each village were considered for the study through universal sampling. As the observations under each district were independently selected and there is no intra-cluster correlation or unequal selection probability, so there is no requirement for clustering or sampling design adjustment.

**Table 4:** Disease status and their operational definitions for the study.

Attributes	Subgroups	Operational definition
TB Status	Currently receiving ATT	Anyone who claims to have been diagnosed with TB and/or currently receiving ATT.
	Completed ATT within last 2 years	Anyone who claims to have been diagnosed and/or treated for TB in the past 2 years.
	Old TB	Anyone who claims to have been diagnosed and/or treated for TB within last 2 years.
	No TB	Anyone who claims to have never been diagnosed and/or treated for TB.
HIV	Reactive	Participant (or household respondent) reported a formal diagnosis of HIV by a medical practitioner and/or receipt of treatment for HIV.
	Non-reactive	The respondent affirmatively stated that the household member had never been diagnosed with or treated for HIV.
Diabetes	Yes	Participant (or household respondent) reported a formal diagnosis of diabetes by a medical practitioner and/or receipt of treatment for diabetes.
	Don’t Know	The respondent explicitly stated that they did not know whether the household member referred to had diabetes.
	No	The respondent affirmatively stated that the household member had never been diagnosed with or treated for diabetes.
Silicosis	Yes	Participant (or household respondent) reported a formal diagnosis of silicosis by a medical practitioner and/or receipt of treatment for silicosis.
	Don’t Know	The respondent explicitly stated that they did not know the status of silicosis diagnosis in family member referred to.
	No	The respondent affirmatively stated that the household member had never been diagnosed with or treated for silicosis.
Silica Exposure	Exposed to mine work	Anyone who claims to be employed in mining and is exposed to silica dust.
	Exposed in building construction work	Anyone who claims to be exposed to silica dust during work at construction sites.
	No Exposure	Anyone who claims to have never been exposed to silica dust.
Smokers	Current Smoker	Anyone who currently has a smoking habit.
	Past Smoker	Anyone who used to smoke in the past but is not currently smoking.
	Never a Smoker	Anyone who claims to have never smoked a cigarette.
Complaint of Cough	Cough Present	Anyone who claims to have been suffering from a cough for the past 2 weeks.
	No Cough	Anyone who claims not to have a cough.

**Table 5:** Prevalence of TB and vulnerable groups along with respective 95% Confidence Intervals (n = 82636).

TB & Vulnerable groups	Prevalence n (%)	95% CI
Tuberculosis	128 (0.155)	0.130 to 0.184
HIV	717 (0.868)	0.8067 to 0.9332
Diabetes	1144 (1.384)	1.307 to 1.466
Silicosis	990 (1.198)	1.126 to 1.275
Silica Exposure	910 (1.101)	1.032 to 1.175
Smokers	3888 (4.705)	4.56 to 4.851
Complaint of Cough	3414 (4.131)	3.998 to 4.269

Table 6 is representing the stratified distribution of prevalence among the various categories of the vulnerable groups. It portrays that study participants belonging to the respective exposed groups of the vulnerable population have higher prevalence of Tuberculosis. The smokers recorded the highest prevalence of TB (Past smokers: 1.07%; Current Smokers: 0.36%), followed by silica exposure among mine workers and construction site workers showing a remarkably higher prevalence of TB (Exposure at mines:0.32%; Exposure at building construction: 0.67%). People with HIV also have a higher TB prevalence (0.56%) than the non-reactive group (0.15%). While the participants with diabetes mellitus and cough greater than 2 weeks had TB prevalence of 0.35% and 0.29% respectively. Lower prevalence was observed under the respective unexposed group, suggesting positive association between the vulnerabilities and TB prevalence.

**Table 6:** Distribution of TB status by vulnerable groups (Crude analysis).

Vulnerable groups	Categories	Total(n)	TB cases^*^	Prevalence (%)
HIV	Reactive	717	4	0.56
	Non-Reactive	81919	124	0.15
Diabetes	Yes	1144	4	0.35
	Don’t Know	1694	2	0.12
	No	79798	122	0.15
Silicosis	Yes	990	1	0.10
	Don’t Know	1116	1	0.09
	No	80530	126	0.16
Silica Exposure	Exposed to Mine Work	312	1	0.32
	Exposed to Building Construction	598	4	0.67
	No Exposure	81726	123	0.15
Smoker	Current Smoker	3888	14	0.36
	Past Smoker	841	9	1.07
	Never a Smoker	77907	105	0.13
Cough	Cough Present	3414	10	0.29
	No Cough	79222	118	0.15

*TB cases involved the two categories of TB status including “Completed ATT within the last two years” and “Currently receiving ATT”.

The results show a small absolute number of events in the silicosis, silica-exposure and HIV-reactive strata. These subgroups contain very small absolute numbers of TB cases, resulting in wide confidence intervals. The estimates found in the study must therefore be regarded as exploratory and hypothesis-generating rather than definitive.

Table 7 describes the prevalence of TB among HIV positives individuals from different studied districts. It is evident from the available data that each district had around 1% TB prevalence among its HIV positive individuals. However, among these districts, Pune reported highest 1.25% prevalence of TB, whereas Jaipur, Lucknow and Patna recorded least 0% prevalence of TB among HIV positive individuals.

**Table 7:** The prevalence of TB in the districts among the HIV positive cases.

District	Total individuals	HIV reactive (n)	TB cases among HIV + ve (n)	TB Prevalence among HIV + ve (%)
Bhopal	19372	168	1	0.60
Jaipur	13122	4	0	0.00
Lucknow	20222	0	0	0.00
Patna	3033	5	0	0.00
Pune	12537	80	1	1.25
Ratlam	14350	460	2	0.43
Total	82636	717	4	0.56

## Discussion

The study conducted here is a large-scale community-based cross-sectional survey which covers a total population of 82,636 covering six districts across five states. It gives us the frequencies and percentages of the various population parameters as also the prevalence of various comorbidities of importance highlighting the distribution of Tuberculosis and TB vulnerable population.

The previous studies were different in their outlook with relatively smaller sample size that failed to provide a bigger picture of the TB vulnerability scenario in India [12]. Our study covers a sizeable population of 82,636 over a varied geography covering six districts in five states that provides us with a perspective as to which groups require special attention and gives us a measurable estimate to work on the same. Moreover, the data highlighted in the study presents the current scenario. The results section portrays population characteristics along with the self-reported TB status of the individuals that highlights the population health gaps and prompts interventions to overcome the situation. This data is of importance for conducting collaborative activities as suggested by the NTEP guidelines for HIV positive cases and Diabetes. This can be effective baseline for ground level work in the active case finding and suggest individuals that should definitely be tested for TB whenever one of the vulnerabilities is present in that person as suggested by many collaborating guidelines of NTEP. Thus this data is of great value for the district/state TB officers to guide the ACF strategies.

The data shows that the TB prevalence of around 150 per one lakh population. While the national tuberculosis prevalence survey conducted in India from 2019–2021 gave a prevalence of 300 per lakh population. The study data is half of the actual value due to the self-reporting nature of the study; moreover, proxy household reporting has further compounded lower reporting counts. This may also be due to the migration of labourers elsewhere who could not be considered or due to missed cases by chance as well as the result of not microbiologically determining the TB status. The lower number of self-reporting may also be due to stigma related to TB status or identification in the community as a TB-infected household. In another study portraying self-reported cases, it was observed that in western India, the tribal population had a self-reported PTB prevalence of 153 per 100,000 population, which was the least.[13] HIV prevalence report of 2023 stated that HIV incidence per 1000 uninfected population in India was 0.05 (India HIV report 2023). But from the study we get the HIV prevalence to be as high as 8.75 PLHIV per 1000 uninfected individuals. This shows that HIV patients have a higher observed prevalence of TB. Among the samples studied, Bhopal, Jaipur, Patna, Pune and Ratlam reported prevalence at 8.74, 0.30, 1.65, 6.42 and 33.12 per 1000 uninfected individuals respectively. It is important to consider that Ratlam and Bhopal show high prevalence which are both in the state of Madhya Pradesh. According to a study, Mizoram being a state dominated by tribal population groups shows a HIV prevalence of 2.3% [14]. Tribal populations generally show a higher prevalence of HIV and other sexually transmitted diseases. Thus the high reporting at specific sites may be due to the rural population having scheduled tribes and other local factors. The prevalence of smokers in the study was found to be 5%, which showed the present smokers were five times the value of past smokers. According to study conducted previously, the percentage of smokers were 15.6% among the Indian demography [8]. The reason lies in the study being based out of community backdrop and reporting by a representative of the household who might not have true knowledge about the smoking status of other members of the family. Moreover a study comparing self-reported smoking status and verification with urine cotinine strip test showed marked discrepancy in the results among adolescents [15].The silica industry in North India is disorganised and profit-driven [16]. General burden of silicosis in our study population was found to be 1.2%. In a previous study it was found that 9.3% of the screened mine workers had silicosis [16]. This study bridges scarcity in data portraying the prevalence of silicosis among the general population. This better equips the Tuberculosis programme for focussed case identification and also nudges the authority for better management of the silicosis cases. Near about 1.1% of the population is occupationally exposed to silica dust in our survey. This data provides us with the information as to where to target efforts that can effectively prevent silicosis and tuberculosis through timely screening and TB preventive treatment. In a study it was noted that tuberculosis in silicotic individuals was around 40.99 per 100 observations and silico-tuberculosis was found in 8.74 per 100 among silicotic individuals [11]. Diabetes had a prevalence of 1.4% at the study sites. Literature search on self-reported studies showed that there is a correlation between self-reported diabetes and socio-economic status (SES). According to a study people of lower SES had a self-reported prevalence of 1.5% which increased to 1.9% to 2.5% in people with the highest level of education.[17] While a study from Haryana gave a number of 9.5% prevalence of DM among the rural population [6]. This large variation in results may be due to the employment of a different methodology in the research study. This might prompt further study in this aspect. People with weak immune systems due to diabetes have greater chance of developing active TB from latent TB infection [18]. That is why programmatic implications for better screening of diabetic patients for Tuberculosis and providing them with TB preventive treatment are important, along with better management of uncontrolled diabetes under the NPCDCS Programme [19]. A persistent cough of more than two weeks is considered a case of presumptive TB until proved otherwise. The result shows 4% of the study population had persistent cough of greater than 2 weeks. This number warrants further investigation into the TB status of these individuals, which can give a clear picture regarding the TB status of the whole population. Though nationwide data on the exact cough prevalence is absent cough over two weeks has a higher sensitivity and lower specificity as a diagnostic tool [9].

NTEP has designated TB-vulnerable groups as the ‘Priority Population’. It includes smokers, silicosis, construction site and mine workers, as well as individuals having HIV, diabetes, and individuals having chronic cough [20,21]. NTEP suggests ACF strategies should prioritise risk groups in order to maximise the diagnosis outcome. This study directly gives the values suggesting the approximate prevalence of ‘priority population’ considered in the study. The households self-reporting with the disease or risk factors can be further followed up for diagnostic confirmation of their respective disease status and streamlined into the district/state healthcare system. Thus our study delves into the dimension of the known TB status and associated vulnerabilities among the study population completely left to the recall capabilities of the participants. This kind of study explores a gap in the diagnostically reported rate and rate as assumed by the participants. In case of TB-HIV cases, NTEP suggests PITC among presumptive TB cases; early initiation of ART among HIV infected individuals; Nutritional support for TB and HIV patients. In consideration of TB and diabetes bidirectional screening is suggested. Thus our study is very much aligned with the objectives of the NTEP and the results obtained enables in achieving the same. Certain programmatic recommendations like systematic screening of current and past smokers especially those with cough under routine NTEP active case finding campaigns and inclusion of workers with known occupational silica exposure like mine and construction workers as a priority population for annual TB screening and consideration of TB preventive treatment may also be suggested in line with WHO and NTEP silicosis-TB guidelines.

Thus, summarising the prevalence data on TB, current smokers, silicosis, silica exposure, diabetes mellitus and complaint of cough was 150 per one lakh population, 5%, 1.2%, 1.1%, 1.4% and 4% respectively. The data obtained in this study consistently recorded a low prevalence rate with respect to the TB vulnerabilities as reported in the previous studies. It is emphasized in the study that stratified estimates for silicosis, silica-dust exposure and HIV-TB co-prevalance rest on very small number of data. This resulted in wide confidence intervals indicating substantial uncertainty; these estimates should therefore be considered as hypothesis-generating instead of confirmatory. The reason behind such outcome might lie in the methodology employed for conducting this study. But the study provides us with a broad context estimates in a large and varied population and also provides us with an eagle eye view of the health status and indirectly the awareness of the populace regarding their own health conditions. Larger multi-centric studies having microbiological verification and subsequent follow-up are required to refine the estimates and further assess their public-health significance.

### Strengths and limitations

Strengths: This study involves extensive multi-district multi-state sample of 82,636 individuals from six districts of different Indian regions, achieved through universal household enumeration and rigorous data cleaning, offering robust, community-level insights into rural TB vulnerabilities. Face-to-face interviews were conducted by trained field staff in local languages, capturing real-world data on key risk factors (HIV, diabetes, silicosis, smoking, chronic cough) often underrepresented in routine surveillance. This study provides practical, evidence-based findings to inform targeted NTEP strategies, comorbidity screening, occupational interventions, and equitable TB control across varied settings. There have been previous studies exploring self-reported levels and socio-economic patterns in the distribution of TB which highlight the major social backdrop of the TB disease.[23]

Limitations: The true-burden of TB is substantially underestimated in self-reported TB prevalence studies conducted in Indian household surveys. NFHS estimates around 304 per 100,000 while national prevalence surveys show a prevalence-to-notification ratio of ~2.84 and ~64% of symptomatic individuals not seeking care [23]. Almost 15–23% of the adults prefer keeping a family member’s TB status secret due to stigma and is further compounded in this study by proxy reporting from a single household respondent, which introduces knowledge gaps and deliberate non-disclosure that misclassify both TB and comorbidities [24]. This factors provide the indication for future studies that link surveys to Nikshay for verification (where around 24% of cases have gone unmatched in previous exercises) and in a similar way incorporating microbiological confirmation would yield more accurate, bias-adjusted estimates that is essential for targeting the hidden burden [25].

## Conclusion

The management of Tuberculosis requires a lot of planning due to its overlapping with social determinants of health. The mapping of the TB vulnerable population helps us in targeting the exact number of the population who might be at risk of developing tuberculosis which helps in breaking the chain of transmission through active case finding and TB preventive therapy at a very early stage. Thus the result of this study can substantially help in establishing a TB protective shield in the population under consideration.
