# Supplementary material for: Self-reported prevalence of tuberculosis and selected associated vulnerability factors among rural populations in six districts of India: A community-based cross-sectional study
Source: PLoS One. 2026 Oct 16;21(10):e0360758. doi: 10.1371/journal.pone.0360758 (PMC13674778; doi:10.1371/journal.pone.0360758)
Supplement: Supplementary file 1 [file pone.0360758.s001.docx]

# PLOS One

**Self-reported Prevalence of Tuberculosis and Selected Associated Vulnerability Factors among Rural Populations in Six Districts of India: A Community-Based Cross-Sectional Study**

--Manuscript Draft--

| **Manuscript Number:** | PONE-D-26-27950 |
| --- | --- |
| **Article Type:** | Research Article |
| **Full Title:** | Self-reported Prevalence of Tuberculosis and Selected Associated Vulnerability Factors among Rural Populations in Six Districts of India: A Community-Based Cross-Sectional Study |
| **Short Title:** | Self-reported TB prevalence and vulnerability factors in rural India: A Cross-sectional study |
| **Corresponding Author:** | P K Anand  ICMR - National Institute of Health Research Jodhpur, Rajasthan INDIA |
| **Keywords:** | Self-reported; Tuberculosis; Vulnerable populations; HIV; Diabetes; Silicosis; Smokers; Rural population; India |
| **Abstract:** | Background: Tuberculosis is a major public health challenge in India, contributing to one-fourth of global burden of TB disease. Comorbidities in uninfected individuals make them vulnerable to TB disease. This research body studies the prevalence of TB status, PLHIV, Current Smokers, Silicosis, Occupational exposure to silica dust, Diabetes Mellitus and Cough of greater than 2 weeks among the rural population. This focuses on categorising the TB-vulnerable groups in India for better NTEP outcomes. Methods: The study conducted was a community-based cross-sectional survey carried out across six districts of India. Two blocks from each district was selected randomly followed by randomly selected villages under each block. A universal sampling technique was followed for including all the households of the randomly selected villages. The data were collected through a pre-tested semi-structured questionnaire schedule via face-to-face interviews at all the eligible households of the study sites.  Descriptive analysis was performed, and the results were obtained in the form of frequencies and percentages.  Results: The ultimate data consisted of 82,636 individuals, with males at 52% and females at 47%. 77% of the study population were 15 years of age or older. This study analyzes a large dataset. The TB prevalence was around 150 per lakh of the population, while the prevalence of HIV-reactive individuals, current smokers, silicosis, occupational exposure to silica dust, diabetes mellitus and cough of greater than two weeks is found in 0.9%, 5%, 1.2%, 1.1%, 1.4% and 4% respectively.  Discussion and Conclusion: The study highlights TB status and selected TB-vulnerable groups. The study has a large sample size, and it follows multipronged approach to study selected vulnerable population groups. This data may improve targeted active case finding among the high-risk groups as outlined by NTEP and provide better TB control strategy outcome at high TB burden settings. |
| **Order of Authors:** | P K Anand |
|  | Manjula Singh |
|  | Purbita Banerjee |
|  | Seema Sahay |
|  | Sampada Bangar |
|  | Braj Ghosh |
|  | Manjeet Chalga |
|  | Anurag Pappu |
|  | VNR Das |
|  |  |

|  | Sarika Gupta |
| --- | --- |
|  | Amber Kumar |
|  | Dhruvendra Pandey |
| **Additional Information:** |  |
| **Question** | **Response** |
| **Financial Disclosure**  Enter a financial disclosure statement that describes the sources of funding for the work included in this submission. Review the [submission guidelines](http://journals.plos.org/plosone/s/submission-guidelines#loc-financial-disclosure-statement) for detailed requirements. View published research articles from [*PLOS ONE*](https://journals.plos.org/plosone/) for specific examples.  This statement is required for submission and **will appear in the published article** if the submission is accepted. Please make sure it is accurate. | Yes |
| **Funded studies**  Enter a statement with the following details:   - Initials of the authors who received each award - Grant numbers awarded to each author - The full name of each funder - URL of each funder website - Did the sponsors or funders play any role in the study design, data collection and analysis, decision to publish, or preparation of the manuscript? |  |
| Did you receive funding for this work? |  |
| Please add funding details.  as follow-up to "**Financial Disclosure**  Enter a financial disclosure statement that describes the sources of funding for the work included in this submission. Review the [submission guidelines](http://journals.plos.org/plosone/s/submission-guidelines#loc-financial-disclosure-statement) for detailed requirements. View published research articles from [*PLOS ONE*](https://journals.plos.org/plosone/) for specific examples.  This statement is required for submission and **will appear in the published article** if | This study was funded by the Indian Council of Medical Research vide File No. 5/8/5/44/ITRC/Implementation/2021/ECD-I. |

| the submission is accepted. Please make sure it is accurate. |  |
| --- | --- |
| **Funded studies**  Enter a statement with the following details:   - Initials of the authors who received each award - Grant numbers awarded to each author - The full name of each funder - URL of each funder website - Did the sponsors or funders play any role in the study design, data collection and analysis, decision to publish, or preparation of the manuscript? |  |
| Did you receive funding for this work?" |  |
| Please select the country of your main research funder (please select carefully as in some cases this is used in fee calculation).  as follow-up to "**Financial Disclosure**  Enter a financial disclosure statement that describes the sources of funding for the work included in this submission. Review the [submission guidelines](http://journals.plos.org/plosone/s/submission-guidelines#loc-financial-disclosure-statement) for detailed requirements. View published research articles from [*PLOS ONE*](https://journals.plos.org/plosone/) for specific examples.  This statement is required for submission and **will appear in the published article** if the submission is accepted. Please make sure it is accurate. | INDIA - IN |
| **Funded studies**  Enter a statement with the following details:   - Initials of the authors who received each award - Grant numbers awarded to each author - The full name of each funder - URL of each funder website - Did the sponsors or funders play any role in the study design, data collection and analysis, decision to publish, or preparation of the manuscript? |  |
|  |  |

| Did you receive funding for this work?" |  |
| --- | --- |
| **Competing Interests**  Use the instructions below to enter a competing interest statement for this submission. On behalf of all authors, disclose any [competing interests](http://journals.plos.org/plosone/s/competing-interests) that could be perceived to bias this work—acknowledging all financial support and any other relevant financial or non-financial competing interests.  This statement is required for submission and **will appear in the published article** if the submission is accepted. Please make sure it is accurate and that any funding sources listed in your Funding Information later in the submission form are also declared in your Financial Disclosure statement.  View published research articles from  [*PLOS ONE*](http://journals.plos.org/plosone/) for specific examples. | The authors have declared that no competing interests exist |
| **NO authors have competing interests**  Enter: *The authors have declared that no competing interests exist.*  **Authors with competing interests**  Enter competing interest details beginning with this statement:  *I have read the journal's policy and the authors of this manuscript have the following competing interests: [insert competing interests here]* |  |
| * typeset |  |
| **Ethics Statement**  Enter an ethics statement for this submission. This statement is required if the study involved:   - Human participants - Human specimens or tissue | The studies involving humans were approved by Institute Ethics  Committee of ICMR - National Institute for Implementation Research on Non-communicable Diseases. The studies were conducted in accordance with the local legislation and institutional requirements.  The participants provided their written informed consent to participate in this study. |

| - Vertebrate animals or cephalopods - Vertebrate embryos or tissues - Field research   Write "N/A" if the submission does not require an ethics statement.  General guidance is provided below. Consult the [submission guidelines](http://journals.plos.org/plosone/s/submission-guidelines#loc-guidelines-for-specific-study-types) for detailed instructions. **Make sure that all information entered here is included in the Methods section of the manuscript.** |  |
| --- | --- |

| **Format for specific study types**  **Human Subject Research (involving human participants and/or tissue)**   - Give the name of the institutional review board or ethics committee that approved the study - Include the approval number and/or a statement indicating approval of this research - Indicate the form of consent obtained (written/oral) or the reason that consent was not obtained (e.g. the data were analyzed anonymously)   **Animal Research (involving vertebrate animals, embryos or tissues)**   - Provide the name of the Institutional Animal Care and Use Committee (IACUC) or other relevant ethics board that reviewed the study protocol, and indicate whether they approved this research or granted a formal waiver of ethical approval - Include an approval number if one was obtained - If the study involved *non-human primates*, add *additional details* about animal welfare and steps taken to ameliorate suffering - If anesthesia, euthanasia, or any kind of animal sacrifice is part of the study, include briefly which substances and/or methods were applied   **Field Research**  Include the following details if this study involves the collection of plant, animal, or other materials from a natural setting:   - Field permit number - Name of the institution or relevant body that granted permission |  |
| --- | --- |
|  |  |
| **Data Availability**  Before publication, Authors are required to make fully available and without restriction all data underlying their findings. Sharing the data underlying your findings increases the visibility, credibility, and impact of your work, while supporting transparency and future scientific discovery. Please see our [PLOS Data](https://journals.plos.org/plosone/s/data-availability) | Data is available with corresponding author and will be provided on request |

| [Policy page](https://journals.plos.org/plosone/s/data-availability) for detailed information on this policy.  A **Data Availability Statement**, detailing where the data can be accessed, is required at first submission. Insert your Data Availability Statement in the box below.  Please see the [data reporting](https://journals.plos.org/plosone/s/submission-guidelines#loc-data-reporting) section of our submission guidelines for instructions on what you need to include in your Data Availability Statement. Examples of acceptable Data Availability Statements are included here for reference:   1. The minimal data set [and accompanying code, where generated] is available at [repository name] via [DOI hyperlink] 2. No data was generated by this study. The following existing data sources were used: [dataset name] from [dataset location] available via [web address or DOI hyperlink]. 3. For Study Protocols: No datasets were generated or analysed during the current study. All relevant data from this study will be made available upon study completion.   *PLOS allows rare exemptions to address legal and ethical concerns. If you have legal or ethical restrictions, please use the box below to detail these in full sentences for the Journal team to consider.*  This statement will be typeset if the manuscript is accepted for publication. |  |
| --- | --- |

<https://www2.cloud.editorialmanager.com/pone/download.aspx?id=44902860&guid=6602396d-ccee-4dee-b7d1-4fb5312ae621&scheme=1>Manuscript	[Click here to](https://www2.cloud.editorialmanager.com/pone/download.aspx?id=44902860&guid=6602396d-ccee-4dee-b7d1-4fb5312ae621&scheme=1)

[access/download;Manuscript;Final_PLOS_One.docx](https://www2.cloud.editorialmanager.com/pone/download.aspx?id=44902860&guid=6602396d-ccee-4dee-b7d1-4fb5312ae621&scheme=1)

1 Title Page
2 **Title:** Self-reported Prevalence of Tuberculosis and Selected Associated
3 Vulnerability Factors among Rural Populations in six Districts of India: A Community-
4 Based Cross-Sectional Study
5 **Authors:** P.K. Anand^1^; Manjula Singh^2^; Purbita Banerjee^3^; Seema Sahay^4^; Sampada
6 Bangar^4^; Braj Raj Ghosh^2^; Manjeet Singh Chalga^2^; Anurag Pappu^5^; VNR Das^5^; Sarika
7 Gupta^6^; Amber Kumar^7^; Dhruvendra Pandey^8^ & *NYKS Research Group*
8 **Corresponding Author:** P.K. Anand, ICMR – National Institute of Health Research,
9 Jodhpur – 342005. E-mail: [ananddmrcjodhpur@gmail.com](mailto:ananddmrcjodhpur@gmail.com)

##### 10 Affiliations:

11 1. ICMR – National Institute of Health Research, Jodhpur – 342005
12 2. Indian Council of Medical Research (ICMR), Ministry of Health &
13 Family Welfare, Government of India
14 3. Indian Institute of Public Health, Gandhinagar, Gujrat
15 4. ICMR – National AIDS Research Institute, Pune
16 5. ICMR – Rajendra Memorial Research Institute on Medical
17 Sciences, Patna
18 6. King George Medical University, Lucknow
19 7. AIIMS, Bhopal
20 8. Government Medical College, Ratlam
21 **Type of Article:** Research Article

##### 22 Word Count:

23 1. Abstract: 296
24 2. Manuscript: 3526

##### 25 No. of figures: 1

26 **No of Tables:** 6
27 **Acknowledgement:** Authors are thankful to Dr Balram Bhargav, Former Director
28 General, Indian Council of Medical Research, New Delhi and Dr. Rajeev Bahal,
29 Director General, Indian Council of Medical Research, New Delhi for their
30 administrative help they provided in execution of this study.
31 **Funding	Information:**	Funded	by	ICMR	vide	File	No.	5/8/5/44/ITRC/
32 Implementation/2021/ECD-I.

33

34 Self-reported Prevalence of Tuberculosis and Selected
35 Associated Vulnerability Factors among Rural Populations in
36 Six Districts of India: A Community-Based Cross-Sectional
37 Study

38

### 39 ABSTRACT

40 **Background**: Tuberculosis is a major public health challenge in India, contributing to one-
41 fourth of global burden of TB disease. Comorbidities in uninfected individuals make them
42 vulnerable to TB disease. This research body studies the prevalence of TB status, PLHIV,
43 Current Smokers, Silicosis, Occupational exposure to silica dust, Diabetes Mellitus and Cough
44 of greater than 2 weeks among the rural population. This focuses on categorising the TB-
45 vulnerable groups in India for better NTEP outcomes.
46 **Methods**: The study conducted was a community-based cross-sectional survey carried out
47 across six districts of India. Two blocks from each district was selected randomly followed by
48 randomly selected villages under each block. A universal sampling technique was followed for
49 including all the households of the randomly selected villages. The data were collected through
50 a pre-tested semi-structured questionnaire schedule via face-to-face interviews at all the
51 eligible households of the study sites. Descriptive analysis was performed, and the results were
52 obtained in the form of frequencies and percentages.
53 **Results**: The ultimate data consisted of 82,636 individuals, with males at 52% and females at
54 47%. 77% of the study population were 15 years of age or older. This study analyzes a large
55 dataset. The TB prevalence was around 150 per lakh of the population, while the prevalence of
56 HIV-reactive individuals, current smokers, silicosis, occupational exposure to silica dust,
57 diabetes mellitus and cough of greater than two weeks is found in 0.9%, 5%, 1.2%, 1.1%, 1.4%
58 and 4% respectively.
59 **Discussion and Conclusion**: The study highlights TB status and selected TB-vulnerable
60 groups. The study has a large sample size, and it follows multipronged approach to study
61 selected vulnerable population groups. This data may improve targeted active case finding
62 among the high-risk groups as outlined by NTEP and provide better TB control strategy
63 outcome at high TB burden settings.

### 64 INTRODUCTION

65 TB is the 10^th^ leading cause of death worldwide and 2^nd^ leading cause of death from a single
66 infectious agent worldwide after COVID-19 ^1^. According to the Global TB Report 2025, India
67 accounts for 25% of the Global TB Burden which is the highest in the world^1^. Tuberculosis is
68 directly linked with the social determinants of health. As stated by the India TB Report 2024,
69 TB is highly influenced by socio-economic development as well as health risk factors like HIV
70 status, smoking habits, silicosis, occupational exposure to silica dust, diabetes mellitus and
71 symptoms like persistent cough of more than 2 weeks. In order to better manage the TB
72 epidemic, it is essential to focus on the underlying vulnerabilities among the demographic that
73 is driving the disease transmission.
74 WHO identifies key vulnerable populations as those with increased risk of TB exposure due
75 to their living conditions, such as prisoners and migrants, individuals with weakened immune
76 systems, and those with chronic illnesses. They face barriers to accessing healthcare services
77 and may have poor health-seeking behaviour, which makes early identification and active case
78 finding essential to ensure timely and effective TB care^3^.
79 Tuberculosis is known to affect people with certain comorbidities disproportionately.
80 Individuals who are suffering from Diabetes, People living with HIV (PLHIV), individuals
81 with silicosis, construction site or mine workers having occupational exposure to silica dust as
82 well as chronic smokers are included in this group^2^. The comorbidities may impact TB
83 infection individually or overlap one another to create a much more complicated scenario. Like
84 Diabetes is one of the risk factors of TB, known to downregulate immunity that put diabetics
85 at a three-fold increased risk of developing active TB in comparison to non-diabetics^4^.
86 Moreover Diabetics show complicated TB treatment outcomes as per the India TB Report
87 2024. The mindful focus on diagnosing, detecting and treating DM may have a beneficial
88 impact on the control of the TB epidemic^4^. HIV another risk factor causes immunosuppression
89 leading to twenty times at higher risk of developing TB in comparison to general population as
90 per the India TB Report 2024. HIV is known to be responsible for 1,50,000 deaths due to TB
91 worlwide^1^. Literature also states that silicosis and silica dust exposure is associated with
92 increased risk of tuberculosis^5^. Then again Smoking has been established as an independent
93 risk factor, with suppressive effects of smoke inhalation on the alveolar macrophage function
94 as well as antibiotic resistance^2^.
95 In India, TB is intertwined with socio-economic vulnerabilities and comorbidities, including
96 diabetes, silicosis, occupational exposure to silica dust, smoking, and cough persisting for more
97 than 2 weeks among other factors , which disproportionately affect marginalized and rural
98 populations due to limited healthcare access, stigma, and delayed diagnosis. The incidence of
99 DM is reported to be 9.6% expected to rise to 10.9% by 2045^6^. While a study showed 12.3%
100 of the sandstone mine workers had radiograph indicating silicosis^7^. Though the exact
101 proportion of the population employed by the industry of stone-mines, stone quarry or the
102 construction sites is not known. Data on smoking showed 15.6% prevalence in India with bidi
103 smoking being the commonest form of smoking specifically in rural areas^8^. Cough on the other
104 hand is the cornerstone of TB diagnosis, persistent cough of over 2 weeks have higher
105 sensitivity and lower specificity as per a TB diagnostic tool^9^ while if unnoticed results in higher
106 disease transmission rate^10^. These drivers elevate infection risk, worsen outcomes, and fuel
107 drug resistance, challenging the National TB Elimination Programme's aim to eliminate TB^5^.
108 Thus ground level research is required to counter these social and high risk factors that drive
109 the Indian TB epidemic. Moreover a study also stresses upon enhanced focus on research in
110 India to better understand the conditions which are interlinked in order to launch effective
111 prevention and management strategies^11^. Thus estimating the prevalence of these populations
112 in a specific geographic backdrop can help us map TB propensity of a particular site and take
113 specific measures in advance with respect to the TB preventive measures or bridge the gaps
114 present in current NTEP implementation.
115 This study was undertaken to estimate the prevalence of TB vulnerable population in Northern
116 India.
117 Objectives:
118 •	To estimate the prevalence of the specific selected factors which make people in the
119 study sites vulnerable to TB.
120 •	To describe the socio-demographic characteristics of the rural population under focus
121 like age, gender, occupation, and community group.
122 •	To study the distribution of the selected vulnerability factors across the study population
123 on the basis of their socio-demographic groups.
124 •	To produce grassroots-level evidence for strengthening targeted TB prevention and
125 screening strategies among high-risk groups.

### 126 METHODOLOGY

127 **Study Design and Setting**: The study was a community-based cross-sectional study
128 conducted, in six districts of India selected on the basis of location of the collaborating centres.
129 The study was conducted under the academic and technical supervision of Indian Council of
130 Medical Research- National Institute of Implementation Research (ICMR-NIIRNCD),
131 Jodhpur, Rajasthan.
132 **Sampling Methodology:** In the first stage, six districts included in the study were purposively
133 selected as they were the established operational areas of the collaborating investigators. This
134 provided feasibility for multi-centric fieldwork. These districts were known to have higher TB
135 Burden and delays in care-seeking. In the second stage within each district two blocks were
136 selected randomly . The villages in the third step was selected randomly from each block. This
137 random selection of villages was done in accordance with the simple random sampling as done
138 under the NYKS study^22^. In the fourth and final stage universal sampling was done of all the
139 households under the selected villages villages of the district. The details of the sampling
140 techniques is elaborated in earlier published article.
141 **Study Population**: The study population included all permanent residents residing in the
142 selected study areas/sites belonging to all age groups and genders.

##### 143 Eligibility Criteria of the Respondent:

144 •	All the families who were residing in the study sites were selected on household basis.
145 •	One member from each family was selected as the household respondent for purpose
146 of collecting information.
147 **Sample Size and Sampling Technique**: The final study population comprised approximately
148 82,636 individuals. Universal sampling was employed, wherein all eligible households in the
149 six selected districts were surveyed to achieve complete enumeration and minimize selection
150 bias. Households found locked were revisited twice at different time points.
151 **Study Variables:** The primary outcome was tuberculosis vulnerability, defined as reported by
152 the people of present TB status as well as having diabetes mellitus, HIV infection, silicosis,
153 occupational silica exposure and individuals having smoking habit. Another aspect emphasized
154 in the study is the TB prevalence, which is calculated by adding the new and old cases of TB .
155 New cases were represented by the “Currently receiving ATT” while the old cases were
156 represented by “Completed ATT in the past 2 years”. Adding both of these values give us TB
157 Prevalence. Independent variables included age group, sex, occupation, and community group.
158 **Data Collection Method and Tool**: Data were obtained via household-based face-to-face
159 interviews using a pretested semi-structured	questionnaire administered by trained
160 investigators. A designated adult respondent provided information for all household members
161 in local dialect. The tool, developed from review of relevant literature and national programme
162 guidelines, assessed socio-demographic characteristics, diabetes mellitus, HIV infection, and
163 silicosis or occupational silica exposure and opinion of the investigators of the study and
164 subject experts.
165 **Data Collection period:** Data collection was initiated on 25 April 2023 and ended on 19
166 August 2023.
167 **Data Analysis**: The data was cleaned and the study sites were geographically represented. The
168 categorical data was expressed as percentages and frequencies.
169 **Ethical Considerations**: The study followed the ethical guidelines as prescribed by the Indian
170 Council of Medical Research (ICMR) guidelines 2025. The Institute Ethics Committee of
171 ICMR-NIIRNCD, Jodhpur granted the ethics approval after going over the research protocol
172 (File No. IEC-ICMR-NIIRNCD/2021/25/3). Informed and written consent was obtained from
173 the head of household or available adult family members before registering in study for
174 providing information of his/her all family members.

### 175 RESULTS

176 The initial dataset comprised 84,004. Data cleaning involved removal of blank entries (n =
177 576), records with cough for >2 weeks (n = 112), missing district information (n = 33), and
178 records with unverifiable respondent identification (n = 647). No missing values were observed
179 for block or village variables. The final analytical dataset consisted of 82,636 records and was
180 used for statistical analysis.
181 Table 2 presents the socio-demographic characteristics of the study population. Of the 82,636
182 participants, 52% were male (n = 43,134), 47% female (n = 39,478), and 1% identified as third
183 gender (n = 24). The majority were aged ≥15 years (77%, n = 63,445), while 7% (n = 6,058)
184 were <6 years and 16% (n = 13,133) were aged 6–15 years. Other details about the population
185 are further elaborated in the table.
186 Table 3 presents key clinical, behavioural and occupational characteristics of the study
187 population (N=82,636). Prior tuberculosis was reported by 0.29%of the population, and 0.08%
188 reported recent completion of anti-tubercular treatment and ongoing treatment was found in
189 0.07% of the study sample. TB Prevalence is calculated from the table by adding the values
190 under “Currently receiving ATT” and “Completed ATT within the last 2 years” which gave a
191 total of 0.15%. As TB statuses could not be confirmed through microbiological laboratory
192 testing, it could not be effectively classified into active TB cases and other criteria. So, the
193 above-mentioned two categories were defined according to the self-reporting nature of the
194 study. “Currently receiving ATT” signified active cases as they were receiving therapy, while
195 “Completed ATT within the last 2 years” signified individuals who completed therapy recently
196 but have a chance of relapse. The majority of relapse cases of TB occur in the first two years
197 of treatment completion. HIV reactivity was observed in 0.9% of individuals. The majority
198 were never smokers (94%). Self-reported silicosis (1.2%) and occupational silica exposure
199 (<2%) were also noted among the population. Diabetes was reported by 1.4% and cough was
200 present in 4% of participants.
201 Table 4 tabulates all the operational definitions that has been considered in the study
202 questionnaire for assessing the health status of the individuals through self reporting. While
203 Table 5 tabulates the prevalence, percentages and 95% CI of all the diseases and risk factors
204 considered in the study and give the raw values with approximation. We will notice that
205 adjustment for clustering or sampling design was not done in the study. This is because initially
206 the districts were chosen as per the presence of collaborator centres, followed by selection of
207 two blocks by simple random sampling. Villages under each block were again chosen by simple
208 random sampling. Finally all the households and every member of each village were considered
209 for the study through universal sampling. As the observations under each district were
210 independently selected and there is no intra-cluster correlation or unequal selection probability,
211 so there is no requirement for clustering or sampling design adjustment.
212 Table 6 is representing the stratified distribution of prevalence among the various categories of
213 the vulnerable groups. It portrays that study participants belonging to the respective exposed
214 groups of the vulnerable population have higher prevalence of Tuberculosis. The smokers
215 recorded the highest prevalence of TB (Past smokers: 1.07%; Current Smokers: 0.36%),
216 followed by silica exposure among mine workers and construction site workers showing a
217 remarkably higher prevalence of TB (Exposure at mines:0.32%; Exposure at building
218 construction: 0.67%). People with HIV also have a higher TB prevalence (0.56%) than the non-
219 reactive group (0.15%). While the participants with diabetes mellitus and cough greater than 2
220 weeks had TB prevalence of 0.35% and 0.29% respectively. Lower prevalence was observed
221 under the respective unexposed group, suggesting positive association between the
222 vulnerabilities and TB prevalence.

| **Table 1: Geographical Distribution of the study participants (n=82636):** | | |
| --- | --- | --- |
| **District Name** | **Household No. (%)** | **Individuals Covered No.**  **(%)** |
| Bhopal | 3187 (25%) | 19372 (23.4%) |
| Jaipur | 2169 (17%) | 13122 (15.9%) |
| Lucknow | 3868 (30%) | 20222 (24.5%) |
| Patna | 458 (3.6%) | 3033 (3.7%) |
| Pune | 1087 (8.5%) | 12537 (15.2%) |
| Ratlam | 1967 (15.4%) | 14350 (17.4%) |
| **Total** | **12736 (100%)** | **82636 (100%)** |

223

| **Table 2: Characteristics of the study participants (n=82636):** | | |
| --- | --- | --- |
| **CHARACTERISTICS** | **CATEGORIES** | **No. (%)** |
| **Gender** | Male | 43134 (52%) |
|  | Female | 39478 (47%) |
|  | Third Gender | 24 (<1%) |
|  | **Total** | **82636 (100%)** |
| **Age group** | 0 to 5 years of age | 6058 (7%) |
|  | 6 to 15 years of age | 13133 (16%) |
|  | Greater than 15 years of age | 63445 (77%) |
|  | **Total** | **82636 (100%)** |
| **Occupation** | Agriculture & Allied | 11807 (14%) |

|  | Unskilled/Manual Workers | 5,764 (7%) |
| --- | --- | --- |
|  | Skilled/Semi-skilled Workers | 6,375 (8%) |
|  | Professional workers & Business Owners | 4,186 (5%) |
|  | Not in Labour Force | 54,504 (66%) |
|  | **Total** | **82636 (100%)** |
| **Community Group** | Scheduled Caste | 25200 (30%) |
|  | Scheduled Tribe | 11493 (14%) |
|  | Other Backward Classes | 36073 (44%) |
|  | General | 9870 (12%) |
|  | **Total** | **82636 (100%)** |

224

| **Table 3: Distribution of Tuberculosis and other vulnerable groups among the**  **participants (n=82636)** | | |
| --- | --- | --- |
| **TB and selected vulnerable**  **groups.** | **Sub-groups** | **n (%)** |
| TB status | Currently receiving ATT | 60(0.07%) |
|  | Completed ATT within last 2  years | 68 (0.08%) |
|  | Old TB | 236 (0.29%) |
|  | No TB | 82272 (99.56%) |
|  | **TOTAL** | **82636 (100%)** |
| HIV | Reactive | 717 (0.87%) |
|  | Non-Reactive | 81919 (99.13%) |
|  | **TOTAL** | **82636 (100%)** |

| Diabetes | Yes | 1144 (1.38) |
| --- | --- | --- |
|  | Don’t Know | 1694 (2.05) |
|  | No | 79798 (96.57) |
|  | **TOTAL** | **826362 (100%)** |
| Silicosis | Yes | 990 (1.20%) |
|  | Don’t Know | 1116 (1.35%) |
|  | No | 80530 (97.45%) |
|  | **TOTAL** | 82632 (100%) |
| Silica Exposure | Exposed to mine work | 312 (0.38%) |
|  | Exposed in building  construction work | 598 (0.72%) |
|  | No Exposure | 81726 (98.90%) |
|  | **TOTAL** | **82636 (100%)** |
| Smokers | Current Smoker | 3888 (4.70%) |
|  | Past Smoker | 841 (1.02%) |
|  | Never a Smoker | 77907 (94.28%) |
|  | **TOTAL** | **82636 (100%)** |
| Cough >2 weeks | Cough Present | 3414 (4.13%) |
|  | No Cough | 79222 (95.87%) |
|  | **TOTAL** | **82636 (100%)** |

225

| **Table 4: Disease Status and their operational definitions for the study:** | | |
| --- | --- | --- |
| **Attributes** | **Subgroups** | **Operational Definition** |

| TB Status | Currently receiving ATT | Anyone who claims to have been diagnosed with TB and/or currently receiving ATT. |
| --- | --- | --- |
|  | Completed ATT within last 2 years | Anyone who claims to have been diagnosed and/or treated for TB in the past 2 years. |
|  | Old TB | Anyone who claims to have been diagnosed and/or treated for TB within  last 2 years. |
|  | No TB | Anyone who claims to have never  been diagnosed and/or treated for TB. |
| HIV | Reactive | Anyone who claims to have been  diagnosed with HIV and/or received or is receiving ART. |
|  | Non-reactive | Anyone who have never been  diagnosed and/or treated for HIV. |
| Diabetes | Yes | Anyone who claims to have been  diagnosed and/or treated for diabetes. |
|  | Don’t Know | Anyone who claims not to know their  diabetes status. |
|  | No | Anyone who claims not to have been diagnosed and/or treated for diabetes. |

| Silicosis | Yes | Anyone who claims to have been  diagnosed and/or treated for silicosis. |
| --- | --- | --- |
|  | Don’t Know | Anyone who claims to be unaware if  they have silicosis. |
|  | No | Anyone who claims to have never  been diagnosed and/or treated for silicosis. |
| Silica  Exposure | Exposed to mine  work | Anyone who claims to be employed in  mining and is exposed to silica dust. |
|  | Exposed in building construction work | Anyone who claims to be exposed to silica dust during work at construction  sites. |
|  | No Exposure | Anyone who claims to have never  been exposed to silica dust. |
| Smokers | Current Smoker | Anyone who currently has a smoking  habit. |
|  | Past Smoker | Anyone who used to smoke in the past  but is not currently smoking. |
|  | Never a Smoker | Anyone who claims to have never  smoked a cigarette. |
| Complaint of Cough | Cough Present | Anyone who claims to have been suffering from a cough for the past 2  weeks. |
|  | No Cough | Anyone who claims not to have a  cough. |

226

227

| **Table 5: Prevalence of TB and Vulnerable groups along with respective 95%**  **Confidence Intervals (n = 82636)** | | |
| --- | --- | --- |
| **TB & Vulnerable Groups** | **Prevalence n (%)** | **95% CI** |
| Tuberculosis | 128 (0.155) | 0.130 to 0.184 |
| HIV | 717 (0.868) | 0.8067 to 0.9332 |
| Diabetes | 1144 (1.384) | 1.307 to 1.466 |
| Silicosis | 990 (1.198) | 1.126 to 1.275 |

| Silica Exposure | 910 (1.101) | 1.032 to 1.175 |
| --- | --- | --- |
| Smokers | 3888 (4.705) | 4.56 to 4.851 |
| Complaint of Cough | 3414 (4.131) | 3.998 to 4.269 |

228

| **Table 6. Distribution of TB Status by vulnerable groups (Crude Analysis)** | | | | |
| --- | --- | --- | --- | --- |
| **Vulnerable**  **groups** | **Categories** | **Total(n)** | **TB**  **cases^*^** | **Prevalence (%)** |
| HIV | Reactive | 717 | 4 | 0.56 |
|  | Non-Reactive | 81919 | 124 | 0.15 |
| Diabetes | Yes | 1144 | 4 | 0.35 |
|  | Don’t Know | 1694 | 2 | 0.12 |
|  | No | 79798 | 122 | 0.15 |
| Silicosis | Yes | 990 | 1 | 0.10 |
|  | Don’t Know | 1116 | 1 | 0.09 |
|  | No | 80530 | 126 | 0.16 |
| Silica  Exposure | Exposed to Mine Work | 312 | 1 | 0.32 |
|  | Exposed to Building  Construction | 598 | 4 | 0.67 |
|  | No Exposure | 81726 | 123 | 0.15 |
| Smoker | Current Smoker | 3888 | 14 | 0.36 |
|  | Past Smoker | 841 | 9 | 1.07 |
|  | Never a Smoker | 77907 | 105 | 0.13 |
| Cough | Cough Present | 3414 | 10 | 0.29 |

|  | No Cough | 79222 | 118 | 0.15 |
| --- | --- | --- | --- | --- |
| *TB cases involved the two categories of TB status including “Completed ATT  within the last two years” and “Currently receiving ATT”. | | | | |

229

### 230 DISCUSSION

231 The study conducted here is a large-scale community-based cross-sectional survey which
232 covers a total population of 82,636 covering six districts across five states. It gives us the
233 frequencies and percentages of the various population parameters as also the prevalence of
234 various comorbidities of importance highlighting the distribution of Tuberculosis and TB
235 vulnerable population.
236 The previous studies were different in their outlook with relatively smaller sample size that
237 failed to provide a bigger picture of the TB vulnerability scenario in India ^12^. Our study covers
238 a sizeable population of 82,636 over a varied geography covering six districts in five states
239 that provides us with a perspective as to which groups require special attention and gives us a
240 measurable estimate to work on the same. Moreover, the data highlighted in the study presents
241 the current scenario. The results section portrays population characteristics along with the self-
242 reported TB status of the individuals that highlights the population health gaps and prompts
243 interventions to overcome the situation. This data is of importance for conducting collaborative
244 activities as suggested by the NTEP guidelines for HIV positive cases and Diabetes. This can
245 be effective baseline for ground level work in the active case finding and suggest individuals
246 that should definitely be tested for TB whenever one of the vulnerabilities is present in that
247 person as suggested by many collaborating guidelines of NTEP. Thus this data is of great value
248 for the district/state TB officers to guide the ACF strategies.
249 The data shows that the TB prevalence of around 150 per one lakh population. While the
250 national tuberculosis prevalence survey conducted in India from 2019-2021 gave a prevalence
251 of 300 per lakh population. The study data is half of the actual value due to the self-reporting
252 nature of the study; moreover, proxy household reporting has further compounded lower
253 reporting counts. This may also be due to the migration of labourers elsewhere who could not
254 be considered or due to missed cases by chance as well as the result of not microbiologically
255 determining the TB status. The lower number of self-reporting may also be due to stigma
256 related to TB status or identification in the community as a TB-infected household. In another
257 study portraying self-reported cases, it was observed that in western India, the tribal population
258 had a self-reported PTB prevalence of 153 per 100,000 population, which was the least.^13^ HIV
259 prevalence report of 2023 stated that HIV incidence per 1000 uninfected population in India
260 was 0.05 (India HIV report 2023). But from the study we get the HIV prevalence to be as high
261 as 8.75 PLHIV per 1000 uninfected individuals. This shows that HIV as a high risk group is of
262 utmost importance. Among the samples studied, Bhopal, Jaipur, Patna, Pune and Ratlam
263 reported prevalence at 8.74, 0.30, 1.65, 6.42 and 33.12 per 1000 uninfected individuals
264 respectively. It is important to consider that Ratlam and Bhopal show high prevalence which
265 are both in the state of Madhya Pradesh. According to a study, Mizoram being a state dominated
266 by tribal population groups shows a HIV prevalence of 2.3%^14^. Tribal populations generally
267 show a higher prevalence of HIV and other sexually transmitted diseases. Thus the high
268 reporting at specific sites may be due to the rural population having scheduled tribes and other
269 local factors. The prevalence of smokers in the study was found to be 5%, which showed the
270 present smokers were five times the value of past smokers. According to study conducted
271 previously, the percentage of smokers were 15.6% among the Indian demography^8^. The reason
272 lies in the study being based out of community backdrop and reporting by a representative of
273 the household who might not have true knowledge about the smoking status of other members
274 of the family. Moreover a study comparing self-reported smoking status and verification with
275 urine cotinine strip test showed marked discrepancy in the results among adolescents^15^.The
276 silica industry in North India is disorganised and profit-driven ^16^. General burden of silicosis
277 in our study population was found to be 1.2%. In a previous study it was found that 9.3% of
278 the screened mine workers had silicosis^16^. This study bridges scarcity in data portraying the
279 prevalence of silicosis among the general population. This better equips the Tuberculosis
280 programme for focussed case identification and also nudges the authority for better
281 management of the silicosis cases. Near about 1.1% of the population is occupationally exposed
282 to silica dust in our survey. This data provides us with the information as to where to target
283 efforts that can effectively prevent silicosis and tuberculosis through timely screening and TB
284 preventive treatment. In a study it was noted that tuberculosis in silicotic individuals was
285 around 40.99 per 100 observations and silico-tuberculosis was found in 8.74 per 100 among
286 silicotic individuals^11^. Diabetes had a prevalence of 1.4% at the study sites. Literature search
287 on self-reported studies showed that there is a correlation between self-reported diabetes and
288 socio-economic status (SES). According to a study people of lower SES had a self-reported
289 prevalence of 1.5% which increased to 1.9% to 2.5% in people with the highest level of
290 education.^17^ While a study from Haryana gave a number of 9.5% prevalence of DM among the
291 rural population^6^. This large variation in results may be due to employment of a different
292 methodology in the research study. This might prompt further study in this aspect. People with
293 weak immune systems due to diabetes have greater chance of developing active TB from latent
294 TB infection^18^. That is why programmatic implications for better screening of diabetic patients
295 for Tuberculosis and providing them with TB preventive treatment are important, along with
296 better management of uncontrolled diabetes under the NPCDCS Programme^19^. A persistent
297 cough of more than two weeks is considered a case of presumptive TB until proved otherwise.
298 The result shows 4% of the study population had persistent cough of greater than 2 weeks. This
299 number warrants further investigation into the TB status of these individuals, which can give a
300 clear picture regarding the TB status of the whole population. Though nationwide data on the
301 exact cough prevalence is absent, cough over two weeks has a higher sensitivity and lower
302 specificity as a diagnostic tool^9^.
303 NTEP has designated TB-vulnerable groups as the ‘Priority Population’. It includes smokers,
304 silicosis, construction site and mine workers, as well as individuals having HIV, diabetes, and
305 individuals having chronic cough^20,21^. NTEP suggests ACF strategies should prioritise risk
306 groups in order to maximise the diagnosis outcome. This study directly gives the values
307 suggesting the approximate prevalence of ‘priority population’ considered in the study. The
308 households self-reporting the disease or risk factors can be further followed up for
309 diagnostic confirmation of their respective disease status and streamlined into the district/state
310 healthcare system. Thus, our study delves into the dimension of the known TB status and
311 associated vulnerabilities among the study population completely left to the recall capabilities
312 of the participants. This kind of study explores a gap in the diagnostically reported rate and rate
313 as assumed by the participants. In case of TB-HIV cases, NTEP suggests PITC among
314 presumptive TB cases; early initiation of ART among HIV infected individuals; nutritional
315 support for TB and HIV patients. In consideration of TB and diabetes bidirectional screening
316 is suggested. Thus our study is very much aligned with the objectives of the NTEP and the
317 results obtained enables achieving the same.
318 Thus, summarising the prevalence data on TB, current smokers, silicosis, silica exposure,
319 diabetes mellitus and complaint of cough was 150 per one lakh population, 5%, 1.2%, 1.1%,
320 1.4% and 4% respectively. The data obtained in this study consistently recorded a low
321 prevalence rate with respect to the TB vulnerabilities as reported in the previous studies. The
322 reason behind such outcome might lie in the methodology employed for conducting this study.
323 But the study provides us with a broad context estimates in a large and varied population and
324 also provides us with an eagle eye view of the health status and indirectly the awareness of the
325 populace regarding their own health conditions.

326

### 327 STRENGTHS AND LIMITATIONS

328 Strengths : This study involves extensive multi-district multi-state sample of 82,636 individuals
329 from six districts of different Indian regions, achieved through universal household
330 enumeration and rigorous data cleaning, offering robust, community-level insights into rural
331 TB vulnerabilities. Face-to-face interviews were conducted by trained field staff in local
332 languages, capturing real-world data on key risk factors (HIV, diabetes, silicosis, smoking,
333 chronic cough) often underrepresented in routine surveillance. This study provides practical,
334 evidence-based findings to inform targeted NTEP strategies, comorbidity screening,
335 occupational interventions, and equitable TB control across varied settings. There have been
336 previous studies exploring self-reported levels and socio-economic patterns in the distribution
337 of TB which highlight the major social backdrop of the TB disease.^23^
338 Limitations: Dependence on self-reported information for TB status and comorbidities, which
339 may lead to recall errors, under-reporting, or misclassification without clinical or laboratory
340 confirmation. Moreover due to reporting done by one representative per household it causes
341 compounding of misclassification. Cross-sectional design may underestimate true TB burden
342 due to migration, passive reporting, exclusion of symptomatic cases from main analysis, and
343 lack of active screening. Findings may not fully represent whole India, as they are restricted to
344 the selected districts, and potential under-detection of conditions is likely in areas with poor
345 healthcare access.

### 346 CONCLUSION

347 The management of Tuberculosis requires a lot of planning due to its overlapping with social
348 determinants of health. The mapping of the TB vulnerable population helps us in targeting the
349 exact number of the population who might be at risk of developing tuberculosis.This will help in
350 breaking the chain of transmission through active case finding and TB preventive therapy at a
351 very early stage. Thus the result of this study can substantially help in establishing a TB
352 protective shield in the population under consideration.

## 353 NYKS Research Group

354 **Patna:** Priyanka kumari, Principal Technical Support III,
355 [pihupriyanka182@gmail.com](mailto:pihupriyanka182@gmail.com); Anil Kumar, Senior Technical
356 officer, [anilrmri@gmail.com](mailto:anilrmri@gmail.com); Kundan Kunal, Senior Technician-
357 I, [kkunalicmr@gmail.com](mailto:kkunalicmr@gmail.com)
358 **Bhopal:** Bhavna Dhingra,
359 [Bhavna.pediatrics@aiimsbhopal.edu.in](mailto:Bhavna.pediatrics@aiimsbhopal.edu.in), 7773012600; Shikha
360 Malik, [Shikha.pediatrics@aiimsbhopal.edu.in,](mailto:Shikha.pediatrics@aiimsbhopal.edu.in) 9425018080;
361 Mahesh Maheshwari, [mahesh.pediatrics@aiimsbhopal.edu.in,](mailto:mahesh.pediatrics@aiimsbhopal.edu.in)
362 9425428596; Priyanka, [priyanka.ophtho@aiimsbhopal.edu.in](mailto:priyanka.ophtho@aiimsbhopal.edu.in),
363 9555571355; Ashiq Hussain Magrey, [ashiqmagrey@gmail.com](mailto:ashiqmagrey@gmail.com),
364 7006790181; Manmohan Sahu,
365 [Sahumanmohan72@gmail.com](mailto:Sahumanmohan72@gmail.com), 9584876868; Neerja
366 Vishwanathan, [neerjav005@gmail.com](mailto:neerjav005@gmail.com), 8610853458; Aarti
367 Tiwari, [Aartitiwari517@gmail.com](mailto:Aartitiwari517@gmail.com), 9826400517.
368 **Ratlam:** Dr. Umesh Sinha, Associate Professor, Department of
369 Community Medicine, Dr. Laxminarayan Pandey Government
370 Medical College, Ratlam, [umi_sinha@yahoo.com;](mailto:umi_sinha@yahoo.com) Dr. Smriti
371 Pandey, Associate Professor, Department of Pathology, Dr.
372 Laxminarayan Pandey Government Medical College, Ratlam.
373 [drsmrititiwari05@gmail.com;](mailto:drsmrititiwari05@gmail.com) Ashish Singh Patel, Junior
374 Research fellow, [ashishsinghpatel666@gmail.com;](mailto:ashishsinghpatel666@gmail.com) Mr. Prahlad
375 Singh Paraste, Research Assistant, [psparaste95@gmail.com](mailto:psparaste95@gmail.com)
376 **Jaipur:** Hyder Ali; Ganesh Narayan Chaudhary, Abhishek
377 Meena

378

# 379 Referencees

380 1.	Global Tuberculosis Report 2025, World Health Organization. Accessed January 15,
381 2026. Available on <https://www.who.int/teams/global-programme-on-tuberculosis-and-lung->
382 health/tb-reports/global-tuberculosis-report-2025
383 2.	Litvinjenko S, Magwood O, Wu S, Wei X. Burden of tuberculosis among vulnerable
384 populations worldwide: an overview of systematic reviews. *Lancet Infect Dis*.
385 2023;23(12):1395-1407. doi:10.1016/S1473-3099(23)00372-9
386 3.	World Health Organization. Tuberculosis and vulnerable populations. Accessed
387 February 16, 2026. Available on <https://www.who.int/europe/news-room/fact->
388 sheets/item/tuberculosis-and-vulnerable-populations
389 4.	Jeon CY, Murray MB. Diabetes mellitus increases the risk of active tuberculosis: a
390 systematic review of 13 observational studies. *PLoS Med*. 2008;5(7):e152.
391 doi:10.1371/journal.pmed.0050152
392 5.	Jamshidi P, Danaei B, Arbabi M, Mohammadzadeha B, Khelghatia F, Akbari
393 Aghababa A, et al. Silicosis and tuberculosis: A systematic review and meta-analysis.
394 *Pulmonology*. 2025;31(1):2416791. doi:10.1016/j.pulmoe.2023.05.001
395 6.	Kalra S, Anjana RM, Verma M, Pradeepa R, Sharma N, Deepa M et al. Urban-Rural
396 Differences in the Prevalence of Diabetes Among Adults in Haryana, India: The ICMR-
397 INDIAB Study (ICMR-INDIAB-18). *Diabetes Ther Res Treat Educ Diabetes Relat Disord*.
398 2024;15(7):1597-1613. doi:10.1007/s13300-024-01602-w
399 7.	Dhatrak S, Nandi S. Assessment of silica dust exposure profile in relation to
400 prevalence of silicosis among Indian sandstone mine workers: Need for review of standards.
401 *Am J Ind Med*. 2020;63(3):277-281. doi:10.1002/ajim.23077
402 8.	Jindal SK, Aggarwal AN, Chaudhry K, Chhabra SK, D'Souza GA, Gupta D, et al.
403 Tobacco smoking in India: prevalence, quit-rates and respiratory morbidity. *Indian J Chest*
404 *Dis Allied Sci*. 2006;48(1):37-42.
405 9.	Giridharan P, Inbaraj LR, Frederick A, Selvaraju S, Ramraj B, Thiruvengadam K, et
406 al. Diagnostic accuracy of screening and diagnostic tests used in a state-wide tuberculosis
407 prevalence survey in India. *Sci Rep*. 2025;15(1):9305. doi:10.1038/s41598-025-94346-x
408 10.	Tobin EH, Tristram D. Tuberculosis Overview. In: *StatPearls*. StatPearls Publishing;
409 2026. Accessed March 13, 2026. <http://www.ncbi.nlm.nih.gov/books/NBK441916/>
410 11.	Deepaselvi M, Palaniswamy CA, Mummadisingu SS, Throvagunta SC, Pradyumna K
411 , Anbazhagan R, et al. Prevalence of silicosis, tuberculosis among silicotic individuals, and
412 silico-tuberculosis in India: A systematic review. *Lung India Off Organ Indian Chest Soc*.
413 2025;42(6):526-531. doi:10.4103/lungindia.lungindia_100_25
414 12.	Chauhan A, Parmar M, Dash GC, Solanki H, Chauhan S, Sharma J, et al. The
415 prevalence of tuberculosis infection in India: A systematic review and meta-analysis. *Indian J*
416 *Med Res*. 2023;157(2 & 3):135-151. doi:10.4103/ijmr.ijmr_382_23
417 13. Thomas BE, Thiruvengadam K, Vedhachalam C, A S, Rao VG, Vijayachari P, et al.
418 (2021) Prevalence of pulmonary tuberculosis among the tribal populations in India.
419 PLoS ONE 16(6): e0251519. <https://doi.org/10.1371/journal>. pone.0251519
420 14.	Rao A, Mamulwar M, Shahabuddin SM, Roy T, Lalnuntlangi N, Panda S. HIV
421 epidemic in Mizoram, India: A rapid review to inform future responses. *Indian J Med Res*.
422 2022;156(2):203-217. doi:10.4103/ijmr.ijmr_1453_22

423

#### 15. Manan, Norfazillah Ab; Nawi, Azmawati Mohammed; Ahmad, Norfazilah;

424

#### Hassan, Mohd Rohaizat. Self-reported smoking among adolescents: How

425

#### accurate is it with the urine cotinine strip test?. International Journal of

426

Pediatrics and Adolescent Medicine 7(2):p 78-82, June 2020. | DOI:

427

#### 10.1016/j.ijpam.2019.08.001

428	16.	Govindagoudar MB, Singh PK, Chaudhry D, Chaudhary R, Sachdeva A, Dhankar S,

429	et al. Burden of Silicosis among stone crushing workers in India. *Occup Med*.

430	2022;72(6):366-371. doi:10.1093/occmed/kqab146

431

#### 17. Corsi DJ, Subramanian SV. Association between socioeconomic status

432

#### and self-reported diabetes in India: a cross-sectional multilevel analysis. BMJ

433

#### Open 2012;2: e000895. doi:10.1136/ bmjopen-2012-000895

434 18. National Programme for Prevention and Control of Non Communicable Diseases (NP-
435 NCD) Guidelines | Ministry of Health and Family Welfare, Government of India. Accessed
436 March 5, 2026. Available on <https://www.mohfw.gov.in/?q=en/Major-Programmes/non->
437 communicable-diseases-injury-trauma/Non-Communicable-Disease-II/National-Programme-
438 for-Prevention-and-Control-of-Cancer-Diabetes-Cardiovascular-diseases-and-Stroke-
439 NPCDCS
440 19. Banerjee B. National Programme for Prevention and Control of Cancer, Diabetes,
441 Cardiovascular Diseases and Stroke (NPCDCS). In: *DK Taneja’s Health Policies and*
442 *Programmes in India*. Jaypee Brothers Medical Publishers (P) Ltd.; 2017:429-429.
443 doi:10.5005/jp/books/13071_31
444 20. NTEP-National Tuberculosis Elimination Programme : National Strategic Plan
445 2017-2025 by Govt. of India.
446 21. National Tuberculosis Elimination Programme.
447 22. Anand PK, Singh M, Kumar A, Sahay S, Pandey D, Tiwari H, et al(2026) Active
448 screening of presumptive tuberculosis cases in rural communities through youth club
449 members in India: logic model development and feasibility study. Front. Public
450 Health 13:1713220. doi: 10.3389/fpubh.2025.1713220
451 23. Mazumdar S, Satyanarayana S, Pai M. Self-reported tuberculosis in India:
452 evidence from NFHS-4. BMJ Global Health. 2019;4:e001371.
453 <https://doi.org/10.1136/bmjgh-2018-001371>

Figure	[Click here to access/download;Figure;Figures.docx](https://www2.cloud.editorialmanager.com/pone/download.aspx?id=44896120&guid=c9e0c4c5-add6-4828-8ca3-e20ad2025dcb&scheme=1)[<https://www2.cloud.editorialmanager.com/pone/download.aspx?id=44896120&guid=c9e0c4c5-add6-4828-8ca3-e20ad2025dcb&scheme=1>](https://www2.cloud.editorialmanager.com/pone/download.aspx?id=44896120&guid=c9e0c4c5-add6-4828-8ca3-e20ad2025dcb&scheme=1)

Fig 1: Location of the study sites on the map of India
