# Supplementary material for: Self-reported prevalence of tuberculosis and selected associated vulnerability factors among rural populations in six districts of India: A community-based cross-sectional study
Source: PLoS One. 2026 Oct 16;21(10):e0360758. doi: 10.1371/journal.pone.0360758 (PMC13674778; doi:10.1371/journal.pone.0360758)
Supplement: Peer review history

# Peer review: Decision Letter 0

**DOI:** 10.1371/journal.pone.0360758.r001

**Authors:** Yasir Alvi

3 Aug 2026

Dear Dr. Anand,

Thank you for submitting your manuscript to PLOS ONE. After careful consideration, we feel that it has merit but does not fully meet PLOS ONE’s publication criteria as it currently stands. Therefore, we invite you to submit a revised version of the manuscript that addresses the points raised during the review process.

Please submit your revised manuscript by Sep 02 2026 11:59PM. If you will need more time than this to complete your revisions, please reply to this message or contact the journal office at plosone@plos.org. When you're ready to submit your revision, log on to https://www.editorialmanager.com/pone/ and select the 'Submissions Needing Revision' folder to locate your manuscript file.

- A letter that responds to each point raised by the academic editor and reviewer(s). You should upload this letter as a separate file labeled 'Response to Reviewers'.
- A marked-up copy of your manuscript that highlights changes made to the original version. You should upload this as a separate file labeled 'Revised Manuscript with Track Changes'.
- An unmarked version of your revised paper without tracked changes. You should upload this as a separate file labeled 'Manuscript'.

If you would like to make changes to your financial disclosure, please include your updated statement in your cover letter. Guidelines for resubmitting your figure files are available below the reviewer comments at the end of this letter.

If applicable, we recommend that you deposit your laboratory protocols in protocols.io to enhance the reproducibility of your results. Protocols.io assigns your protocol its own identifier (DOI) so that it can be cited independently in the future. For instructions see: https://journals.plos.org/plosone/s/submission-guidelines#loc-laboratory-protocols. Additionally, PLOS ONE offers an option for publishing peer-reviewed Lab Protocol articles, which describe protocols hosted on protocols.io. Read more information on sharing protocols at https://plos.org/protocols?utm_medium=editorial-email&utm_source=authorletters&utm_campaign=protocols.

As the corresponding author, your ORCID iD is verified in the submission system and will appear in the published article. PLOS supports the use of ORCID, and we encourage all coauthors to register for an ORCID iD and use it as well. Please encourage your coauthors to verify their ORCID iD within the submission system before final acceptance, as unverified ORCID iDs will not appear in the published article. *Only*  the individual author can complete the verification step; PLOS staff *cannot*  verify ORCID iDs on behalf of authors.

We look forward to receiving your revised manuscript.

Kind regards,

Yasir Alvi

Academic Editor

PLOS One

Journal Requirements:

When submitting your revision, we need you to address these additional requirements.

1. Please ensure that your manuscript meets PLOS ONE's style requirements, including those for file naming. The PLOS ONE style templates can be found at

https://journals.plos.org/plosone/s/file?id=wjVg/PLOSOne_formatting_sample_main_body.pdf and https://journals.plos.org/plosone/s/file?id=ba62/PLOSOne_formatting_sample_title_authors_affiliations.pdf

2. One of the noted authors is a group or consortium [NYKS Research Group]. In addition to naming the author group, please list the individual authors and affiliations within this group in the acknowledgments section of your manuscript. Please also indicate clearly a lead author for this group along with a contact email address.

3. In the online submission form, you indicated that data is available with corresponding author and will be provided on request

All PLOS journals now require all data underlying the findings described in their manuscript to be freely available to other researchers, either 1. In a public repository, 2. Within the manuscript itself, or 3. Uploaded as supplementary information.

This policy applies to all data except where public deposition would breach compliance with the protocol approved by your research ethics board. If your data cannot be made publicly available for ethical or legal reasons (e.g., public availability would compromise patient privacy), please explain your reasons on resubmission and your exemption request will be escalated for approval.

4. Thank you for stating the following financial disclosure:

This study was funded by the Indian Council of Medical Research vide File No. 5/8/5/44/ITRC/Implementation/2021/ECD-I.

Please state what role the funders took in the study. If the funders had no role, please state: "The funders had no role in study design, data collection and analysis, decision to publish, or preparation of the manuscript."

If this statement is not correct you must amend it as needed.

Please include this amended Role of Funder statement in your cover letter; we will change the online submission form on your behalf.

5. One of the noted authors is a group or consortium [NYKS Research Group]. In addition to naming the author group, please list the individual authors and affiliations within this group in the acknowledgments section of your manuscript. Please also indicate clearly a lead author for this group along with a contact email address.

6. Please ensure that you refer to Figure 1 in your text as, if accepted, production will need this reference to link the reader to the figure.

7. Please include a caption for figure 1.

8. We note that Figure 1 in your submission contain [map/satellite] images which may be copyrighted. All PLOS content is published under the Creative Commons Attribution License (CC BY 4.0), which means that the manuscript, images, and Supporting Information files will be freely available online, and any third party is permitted to access, download, copy, distribute, and use these materials in any way, even commercially, with proper attribution. For these reasons, we cannot publish previously copyrighted maps or satellite images created using proprietary data, such as Google software (Google Maps, Street View, and Earth). For more information, see our copyright guidelines: http://journals.plos.org/plosone/s/licenses-and-copyright.

We require you to either (1) present written permission from the copyright holder to publish these figures specifically under the CC BY 4.0 license, or (2) remove the figures from your submission:

a. You may seek permission from the original copyright holder of Figure 1 to publish the content specifically under the CC BY 4.0 license.

We recommend that you contact the original copyright holder with the Content Permission Form (http://journals.plos.org/plosone/s/file?id=7c09/content-permission-form.pdf) and the following text:

“I request permission for the open-access journal PLOS ONE to publish XXX under the Creative Commons Attribution License (CCAL) CC BY 4.0 (http://creativecommons.org/licenses/by/4.0/). Please be aware that this license allows unrestricted use and distribution, even commercially, by third parties. Please reply and provide explicit written permission to publish XXX under a CC BY license and complete the attached form.”

Please upload the completed Content Permission Form or other proof of granted permissions as an "Other" file with your submission.

In the figure caption of the copyrighted figure, please include the following text: “Reprinted from [ref] under a CC BY license, with permission from [name of publisher], original copyright [original copyright year].”

b. If you are unable to obtain permission from the original copyright holder to publish these figures under the CC BY 4.0 license or if the copyright holder’s requirements are incompatible with the CC BY 4.0 license, please either i) remove the figure or ii) supply a replacement figure that complies with the CC BY 4.0 license. Please check copyright information on all replacement figures and update the figure caption with source information. If applicable, please specify in the figure caption text when a figure is similar but not identical to the original image and is therefore for illustrative purposes only.

The following resources for replacing copyrighted map figures may be helpful:

USGS National Map Viewer (public domain): http://viewer.nationalmap.gov/viewer/

The Gateway to Astronaut Photography of Earth (public domain): http://eol.jsc.nasa.gov/sseop/clickmap/

Maps at the CIA (public domain): https://www.cia.gov/library/publications/the-world-factbook/index.html and https://www.cia.gov/library/publications/cia-maps-publications/index.html

NASA Earth Observatory (public domain): http://earthobservatory.nasa.gov/

Landsat: http://landsat.visibleearth.nasa.gov/

USGS EROS (Earth Resources Observatory and Science (EROS) Center) (public domain): http://eros.usgs.gov/#

Natural Earth (public domain): http://www.naturalearthdata.com/

9. We note you have included a table to which you do not refer in the text of your manuscript. Please ensure that you refer to Table 1 in your text; if accepted, production will need this reference to link the reader to the Table.

10. If the reviewer comments include a recommendation to cite specific previously published works, please review and evaluate these publications to determine whether they are relevant and should be cited. There is no requirement to cite these works unless the editor has indicated otherwise.

11. Please review your reference list to ensure that it is complete and correct. If you have cited papers that have been retracted, please include the rationale for doing so in the manuscript text, or remove these references and replace them with relevant current references. Any changes to the reference list should be mentioned in the rebuttal letter that accompanies your revised manuscript. If you need to cite a retracted article, indicate the article’s retracted status in the References list and also include a citation and full reference for the retraction notice.

Additional Editor Comments:

Please revised the manuscrip taking these suggestions into consideration:

- The objective in the abstract needs to be revised to focus on TB.
- The objective in the manuscript should also be revised to include the various selected factors, as mentioned in the abstract objectives.
- Under the Study Design section, kindly add the names of the six districts (as provided in Table 1).
- Kindly provide details of the randomization process. There seems to be a discrepancy in the sampling strategy compared with the previously published study. The NYKS study (Ref. 22) does not mention random sampling at most levels. Instead, it states that sampling was done in consultation with the district/state TB officers and villages having active NYKS youth clubs.
- Please use the full form of NYKS when it is first mentioned.
- The statement, "The details of the sampling techniques is elaborated in earlier published article," is not sufficient. Since the sampling methodology is critical for understanding the study, kindly provide a brief description here as well, in addition to citing the previously published article.
- Study Variables: The operational definition of TB prevalence needs reconsideration. Defining TB prevalence as "completed ATT in the past 2 years" is questionable. Using "completed ATT in the past 1 year" may be more appropriate.
- The independent variables (age group, sex, occupation, and community group) are listed, but their use in the results is limited. Consider presenting more analyses using these variables.
- In Tables 2 and 3, kindly place the "Total" row only at the end of each table, instead of after every variable.

Reviewers' comments:

Reviewer's Responses to Questions

**Comments to the Author**

1. Is the manuscript technically sound, and do the data support the conclusions?

Reviewer #1: Yes

Reviewer #2: Partly

**********

2. Has the statistical analysis been performed appropriately and rigorously?

Reviewer #1: No

Reviewer #2: Yes

**********

3. Have the authors made all data underlying the findings in their manuscript fully available?

The PLOS Data policy

Reviewer #1: Yes

Reviewer #2: Yes

**********

4. Is the manuscript presented in an intelligible fashion and written in standard English?

Reviewer #1: No

Reviewer #2: Yes

**********

Reviewer #1: The manuscript addresses an important public health issue by estimating self reported TB prevalence and associated vulnerability factors in rural India. The large sample size 82,636 and community based design are appropriate. However, there are some concerns which need to be addressed:

Operational Definitions: Silicosis:

Don’t Know: Anyone who claims to be unaware if they have silicosis.

No: Anyone who claims to have never been diagnosed and/or treated for silicosis.

It is not clear how the two were differentiated.

Results:

1.TB prevalence: The reliance on self reported TB status without microbiological confirmation introduces significant risk of underreporting and misclassification. The authors have rightly identified – “Stigma, recall bias, and lack of clinical validation may explain why the observed prevalence (0.15%, ~150 per lakh) is lower than national estimates (~210 per lakh, India TB Report 2024)”. It was possible to have verified with Nikshay reporting for the study sites to find out the magnitude of underreporting by the respondents.

2.The stratified distribution of prevalence among the various categories of the vulnerable groups

The authors state” The primary objective of the proposed study is “To estimate the prevalence of the specific selected factors which make people in the 119 study sites vulnerable to TB”. It is also mentioned that” The primary outcome was tuberculosis vulnerability, defined as reported by the people of present TB status as well as having diabetes mellitus, HIV infection, silicosis, occupational silica exposure and individuals having smoking habit”

Table 6 presents prevalence and confidence intervals for comorbidities (specific selected factors. This is a cross-sectional study, and therefore temporality is missing. Consequently, it is not possible to determine whether the selected factors increased vulnerability to TB or whether TB itself led to vulnerability. Nonetheless, inferential statistics should be applied to demonstrate that the odds of the vulnerable population having TB are significantly higher than those without vulnerability factors."

Table 6 could be streamlined by moving operational definitions to supplementary material, and authors could focus on prevalence and stratified TB outcomes.

3.Several categories (e.g., silicosis, occupational exposure) have very low prevalence (<2%). These small numbers need to be interpreted cautiously

4.Cough was present in 4% of participants. It is not mentioned if these people were then tested for TB?

5. The study has reported a variable HIV prevalence “among the samples studied, Bhopal, Jaipur, Patna, Pune and Ratlam reported prevalence at 8.74, 0.30, 1.65, 6.42 and 33.12 per 1000 uninfected individuals respectively”. With such high values compared to the overall prevalence of 0.99 the prevalence of TB in these areas among the HIV positives needs to be separately analysed.

The last objective was Grassroots-level evidence for strengthening targeted TB prevention and screening strategies among high-risk groups:

The program already has Diabetes and TB, HIV and TB bidirectional screening. The recommendation for screening smokers and those having occupational silica exposure is not specified.

The manuscript needs editing for grammar and language.

Reviewer #2: It is a good write up but some points need to be modified as stated in the manuscript.Point wise clarification to be given. The language needs to be modified in certain places and educational status of the study population needs to be included in demographic factors.

**********

what does this mean?). If published, this will include your full peer review and any attached files.

If you choose “no”, your identity will remain anonymous but your review may still be made public.

**Do you want your identity to be public for this peer review?**  For information about this choice, including consent withdrawal, please see our Privacy Policy

Reviewer #1: No

Reviewer #2: No

**********

[NOTE: If reviewer comments were submitted as an attachment file, they will be attached to this email and accessible via the submission site. Please log into your account, locate the manuscript record, and check for the action link "View Attachments". If this link does not appear, there are no attachment files.]

To ensure your figures meet our technical requirements, please review our figure guidelines: https://journals.plos.org/plosone/s/figures

You may also use PLOS’s free figure tool, NAAS, to help you prepare publication quality figures: https://journals.plos.org/plosone/s/figures#loc-tools-for-figure-preparation.

NAAS will assess whether your figures meet our technical requirements by comparing each figure against our figure specifications.

Attachment Submitted filename: PONE-D-26-27950.docx

# Author comment: Author response to Decision Letter 1

**DOI:** 10.1371/journal.pone.0360758.r002

7 Sep 2026

To,

The Editor

PLOS One

Subject: - Submission of responses by the authors on the comments received

Dear Editor,

Please find the list of comments received and responses made in revised manuscript

Reviewer Remarks Author Responses

1. Please ensure that your manuscript meets PLOS ONE's style requirements, including those for file naming. Authors thank the Editor for suggestion. We have carefully reviewed and revised the manuscript to comply with all PLOS ONE formatting and style requirements. The file names have been revised according to the journal requirements.

2. One of the noted authors is a group or consortium [NYKS Research Group]. In addition to naming the author group, please list the individual authors and affiliations within this group in the acknowledgments section of your manuscript. Please also indicate clearly a lead author for this group along with a contact email address. We thank the Editor for pointing this out.

NYKS Research group mentioned as author represents group of project staffs who collected data. These all staffs have left the study sites after completion of project activities and currently their contact details are not traceable. Hence we have removed the “NYKS Research group” from the list of authors.

3. In the online submission form, you indicated that data is available with corresponding author and will be provided on request

All PLOS journals now require all data underlying the findings described in their manuscript to be freely available to other researchers, either 1. In a public repository, 2. Within the manuscript itself, or 3. Uploaded as supplementary information.

This policy applies to all data except where public deposition would breach compliance with the protocol approved by your research ethics board. If your data cannot be made publicly available for ethical or legal reasons (e.g., public availability would compromise patient privacy), please explain your reasons on resubmission and your exemption request will be escalated for approval. We thank the Editor for the insightful suggestion. The following correction may be made in information submitted online:-

“The data has been presented in the manuscript.”

4. Thank you for stating the following financial disclosure:

This study was funded by the Indian Council of Medical Research vide File No. 5/8/5/44/ITRC/Implementation/2021/ECD-I.

Please state what role the funders took in the study. If the funders had no role, please state: "The funders had no role in study design, data collection and analysis, decision to publish, or preparation of the manuscript."

If this statement is not correct you must amend it as needed.

Please include this amended Role of Funder statement in your cover letter; we will change the online submission form on your behalf.

We are grateful to the Journal authorities for pointing out the requirements.

We have amended the Funding/ Financial Disclosure statement as requested in page no.7, line number 152 to 155. The revised text now reads

The phrase “This study was funded by the Indian Council of Medical Research (ICMR) vide File No. 5/8/5/44/ITRC/Implementation/2021/ECD-I. The funders had no role in study design, data collection and analysis, decision to publish, or preparation of the manuscript” has been added in page 7 line No. 152-155.

5. One of the noted authors is a group or consortium [NYKS Research Group]. In addition to naming the author group, please list the individual authors and affiliations within this group in the acknowledgments section of your manuscript. Please also indicate clearly a lead author for this group along with a contact email address. We thank the Editor for pointing this out.

NYKS Research group mentioned as author represents group of project staffs who collected data. These all staffs have left the study sites after completion of project activities and currently their contact details are not traceable. Hence we have removed the “NYKS Research group” from the list of authors.

6. Please ensure that you refer to Figure 1 in your text as, if accepted, production will need this reference to link the reader to the figure. We thank the Editors for raising this important point. Figure 1 is now removed from the manuscript, as the information can be better communicated in text.

7. Please include a caption for figure 1. Figure 1 has been removed; hence, this suggestion is not applicable now.

8. We note that Figure 1 in your submission contain [map/satellite] images which may be copyrighted. All PLOS content is published under the Creative Commons Attribution License (CC BY 4.0), which means that the manuscript, images, and Supporting Information files will be freely available online, and any third party is permitted to access, download, copy, distribute, and use these materials in any way, even commercially, with proper attribution. For these reasons, we cannot publish previously copyrighted maps or satellite images created using proprietary data, such as Google software (Google Maps, Street View, and Earth). For more information, see our copyright guidelines: http://journals.plos.org/plosone/s/licenses-and-copyright.

We require you to either (1) present written permission from the copyright holder to publish these figures specifically under the CC BY 4.0 license, or (2) remove the figures from your submission:

a. You may seek permission from the original copyright holder of Figure 1 to publish the content specifically under the CC BY 4.0 license.

We sincerely acknowledge the important suggestion of kind Editor. Since the Figure 1 is a copyrighted material, hence it has been removed from the manuscript.

9. We note you have included a table to which you do not refer in the text of your manuscript. Please ensure that you refer to Table 1 in your text; if accepted, production will need this reference to link the reader to the Table. We thank the Editor for the insightful input with respect to the manuscript.

Table 1 (Geographical Distribution of the study participants) is now explicitly cited in the Results section of the revised manuscript in page 7, line no. 162 and 163.

10. If the reviewer comments include a recommendation to cite specific previously published works, please review and evaluate these publications to determine whether they are relevant and should be cited. There is no requirement to cite these works unless the editor has indicated otherwise. We thank the Editor for pointing out this important point.

We have thoroughly reviewed and finalised the entire reference list cautiously to ensure robustness of the manuscript. We have not included any such reference based on recommendations of reviewer.

11. Please review your reference list to ensure that it is complete and correct. If you have cited papers that have been retracted, please include the rationale for doing so in the manuscript text, or remove these references and replace them with relevant current references. Any changes to the reference list should be mentioned in the rebuttal letter that accompanies your revised manuscript. If you need to cite a retracted article, indicate the article’s retracted status in the References list and also include a citation and full reference for the retraction notice. We are grateful to the Editors for pointing out this factor regarding the cited references.

No retracted articles were identified in the revised manuscript. All references have been checked for accuracy, completeness and correct formatting according to PLOS ONE style. Minor Corrections (author initials, DOI links, access dates) have been made where required.

Response to Additional Editor Comments

1. The objective in the abstract needs to be revised to focus on TB. We thank the Editor for the insightful observation. We have revised the Background/Objective sentence in the abstract to place tuberculosis firmly at the centre of the study.

The revision as follows has been added to page 1 from line number 8 to 12.

“This study estimates the self-reported prevalence of tuberculosis and selected vulnerability factors (HIV, current smoking, silicosis, occupational silica-dust exposure, diabetes mellitus and persistent cough >2 weeks) among the rural demography of six districts in India, to inform targeted active case finding under the National Tuberculosis Elimination Programme (NTEP).”

2. The objective in the manuscript should also be revised to include the various selected factors, as mentioned in the abstract objectives. We are grateful to the Editor for bringing forth this point.

The objective section in the main manuscript has been rewritten to list the selected vulnerability factors explicitly and to keep the focus on tuberculosis.

The revision has been added to Page no. 5 from line no. 96 to 97 as follows. “Objectives

• To examine the distribution of the selected vulnerability factors across socio-demographic strata.”

3. Under the Study Design section, kindly add the names of the six districts (as provided in Table 1). We thank the Editor for such detailed observation of the manuscript.

The “Study Design and Setting” paragraph now explicitly names the six districts as added in Page 5 line numbers 103 to 104 as follows.

“Bhopal (Madhya Pradesh), Jaipur (Rajasthan), Lucknow (Uttar Pradesh), Patna (Bihar), Pune (Maharashtra) and Ratlam (Madhya Pradesh)”

4. Kindly provide details of the randomization process. There seems to be a discrepancy in the sampling strategy compared with the previously published study. The NYKS study (Ref. 22) does not mention random sampling at most levels. Instead, it states that sampling was done in consultation with the district/state TB officers and villages having active NYKS youth clubs. We appreciate the Editor for pointing this out.

We acknowledge this comment and have further clarified the sampling description.

Sampling methodology is now better clarified on page 5-6 and line Nos. from 109-119.

5. Please use the full form of NYKS when it is first mentioned. We thank the editor for pointing out this detail in the manuscript. The full form Nehru Yuva Kendra Sangathan (NYKS) is now introduced at the first mention in the methods section in page no. 5 line number 114.

6. The statement, "The details of the sampling techniques is elaborated in earlier published article," is not sufficient. Since the sampling methodology is critical for understanding the study, kindly provide a brief description here as well, in addition to citing the previously published article. We are grateful to the editor for pointing out the apparent dearth in the sampling technique description.

A concise but complete description of sampling procedure has been clarified on page 5-6 and line Nos. 109-119. The line "The details of the sampling techniques is elaborated in earlier published article," is removed from the manuscript.

7. Study Variables: The operational definition of TB prevalence needs reconsideration. Defining TB prevalence as "completed ATT in the past 2 years" is questionable. Using "completed ATT in the past 1 year" may be more appropriate. We are thankful to the editor for making this important methodological observation. NTEP guidelines define “Previously treated TB” based on TB treatment in past, without mentioning Nos. of years. Majority of relapses do occur within 2 years of diagnosis and this period has been considered for follow-up surveillance by NTEP. Due to these reasons, we categorised all those who reported for having received previous treatment of TB in to 2 categories as (1) who received within last 2 years and (2) old cases. Therefore, we are bound to retain the original definition.

8. The independent variables (age group, sex, occupation, and community group) are listed, but their use in the results is limited. Consider presenting more analyses using these variables. We thank the editor for pointing out this issue. Independent variables have now analyzed more at page 8 in lines 171-176.

9. In Tables 2 and 3, kindly place the "Total" row only at the end of each table, instead of after every variable.

Response to Reviewer Comments We thank the Editor for putting forward this point.

Tables 2 and 3 have been reformatted. The “Total” row appears only once, at the foot of each table as seen in page no. 9,10, 11 and 12.

Reviewer #1

Operational Definitions: Silicosis:

Don’t Know: Anyone who claims to be unaware if they have silicosis.

No: Anyone who claims to have never been diagnosed and/or treated for silicosis.

It is not clear how the two were differentiated. We are grateful to the Reviewer for putting forward this point.

We have put in the effort to clarify the mentioned distinction in the revised Table 4 on pages 14 & 15.

Results:

1.TB prevalence: The reliance on self reported TB status without microbiological confirmation introduces significant risk of underreporting and misclassification. The authors have rightly identified – “Stigma, recall bias, and lack of clinical validation may explain why the observed prevalence (0.15%, ~150 per lakh) is lower than national estimates (~210 per lakh, India TB Report 2024)”. It was possible to have verified with Nikshay reporting for the study sites to find out the magnitude of underreporting by the respondents. We are thankful to the Reviewer for putting this point forward.

We are too in favour of the point that self-reporting underestimates true prevalence and that linkage with Nikshay would have strengthened the estimate.

We have put an effort to expand the Limitations section to discuss the magnitude of possible under-reporting, the role of stigma and proxy reporting, and the value of future studies that incorporate Nikshay verification or microbiological confirmation.

The following addition was done in page 24-25 from line no. 352 to 362.

“The true-burden of TB is substantially underestimated in self-reported TB prevalence studies conducted in Indian household surveys. NFHS estimates around 304 per 100,000 while national prevalence surveys show a prevalence-to-notification ratio of ~2.84 and ~64% of symptomatic individuals not seeking care23. Almost 15-23% of the adults prefer keeping a family member’s TB status secret due to stigma and is further compounded in this study by proxy reporting from a single household respondent, which introduces knowledge gaps and deliberate non-disclosure that misclassify both TB and comorbidities2. This factors provide the indication for future studies that link surveys to Nikshay for verification (where around 24 % of cases have gone unmatched in previous exercises) and in a similar way incorporating microbiological confirmation would yield more accurate, bias-adjusted estimates that is essential for targeting the hidden burden3.”

2. The stratified distribution of prevalence among the various categories of the vulnerable groups

The authors state” The primary objective of the proposed study is “To estimate the prevalence of the specific selected factors which make people in the 119 study sites vulnerable to TB”. It is also mentioned that” The primary outcome was tuberculosis vulnerability, defined as reported by the people of present TB status as well as having diabetes mellitus, HIV infection, silicosis, occupational silica exposure and individuals having smoking habit”

Table 6 presents prevalence and confidence intervals for comorbidities (specific selected factors. This is a cross-sectional study, and therefore temporality is missing. Consequently, it is not possible to determine whether the selected factors increased vulnerability to TB or whether TB itself led to vulnerability. Nonetheless, inferential statistics should be applied to demonstrate that the odds of the vulnerable population having TB are significantly higher than those without vulnerability factors."

Table 6 could be streamlined by moving operational definitions to supplementary material, and authors could focus on prevalence and stratified TB outcomes. We are grateful to the Reviewer for putting this aspect forward.

We acknowledge the fact that a cross-sectional design is not capable of establishing temporality or causality.

• We have softened all language that could be interpreted as causal (replacing “increased vulnerability”/ “higher risk” with “higher observed prevalence” or “association”). Correction was added in page 21 from line no. 264-265.-This shows that HIV patients have a higher observed prevalence of TB.”

3. Several categories (e.g., silicosis, occupational exposure) have very low prevalence (<2%). These small numbers need to be interpreted cautiously We thank the reviewer for putting forward this point.

We are in agreement that the low prevalence rates noted in several categories needs to be interpreted cautiously.

Corrections were added in the Results Section Page 9 from line 203 to 206 as follows:

“The results show a small absolute number of events in the silicosis, silica-exposure and HIV-reactive strata. These subgroups contain very small absolute numbers of TB cases, resulting in wide confidence intervals. The estimates found in the study must therefore be regarded as exploratory and hypothesis-generating rather than definitive.”

Further caution added in the Discussions Section in page 23 & 24 line no.330-334 and 338 to 339.

“It is emphasized in the study that stratified estimates for silicosis, silica-dust exposure and HIV-TB co-prevalnece rest on very small number of data. This resulted in wide confidence intervals indicating substantial uncertainty; these estimates should therefore be considered as hypothesis-generating instead of confirmatory.”

“Larger multi-centric studies having microbiological verification and subsequent follow-up are required to refine the estimates and further assess its public-health significance.”

4. Cough was present in 4% of participants. It is not mentioned if these people were then tested for TB? We appreciate the Reviewer for the query.

In this study, individuals reporting of cough >2 weeks duration were not subjected to further diagnostic testing, though they were advised to seek health care, as it was beyond the scope of the study.

5. The study has reported a variable HIV prevalence “among the samples studied, Bhopal, Jaipur, Patna, Pune and Ratlam reported prevalence at 8.74, 0.30, 1.65, 6.42 and 33.12 per 1000 uninfected individuals respectively”. With such high values compared to the overall prevalence of 0.99 the prevalence of TB in these areas among the HIV positives needs to be separately analysed. We are grateful to the Reviewer for putting forward this point.

We have added a brief district-wise and HIV-stratified analysis of TB prevalence in Table 7. The results show the numbers of HIV-TB co-prevalent cases as very small and they must be approached with appropriate caution.

The last objective was Grassroots-level evidence for strengthening targeted TB prevention and screening strategies among high-risk groups:

The program already has Diabetes and TB, HIV and TB bidirectional screening. The recommendation for screening smokers and those having occupational silica exposure is not specified.

We thank the reviewer for pointing this out.

The following text has been added in page 23 from line no. 321 to 326 as follows:

“Certain programmatic recommendations like systematic screening of current and past smokers especially those with cough under routine NTEP active case finding campaigns and inclusion of workers with known occupational silica exposure like mine and construction workers as a priority population for annual TB screening and consideration of TB preventive treatment may also be suggested in line with WHO and NTEP silicosis-TB guidelines.”

The manuscript needs editing for grammar and language. We are grateful to the Reviewer for pointing out these deficiencies in the manuscript.

The entire manuscript has been carefully copy-edited for grammar, syntax, clarity and consistency of terminology. A professional language review was performed.

Reviewer #2

It is a good write up but some points need to be modified as stated in the manuscript. Point wise clarification to be given. The language needs to be modified in certain places and educational status of the study population needs to be included in demographic factors. We thank the Reviewer for the overall positive assessment. All specific points marked in the manuscript have been addressed. Language and grammar have been thoroughly revised throughout. Educational status was not collected in the original questionnaire; we have therefore noted this as a limitation and have recommended its inclusion if any such survey is conducted in the future. The socio-demographic table already present age, sex, occupation and community group; these remain the primary stratification variables in the study.

Please consider the responses for evaluating the revised manuscript.

Thanking You

Yours

Dr P.K. Anand

Attachment Submitted filename: Response to reviewers.docx

# Peer review: Decision Letter 1

**DOI:** 10.1371/journal.pone.0360758.r003

**Authors:** Yasir Alvi

30 Sep 2026

Self-reported Prevalence of Tuberculosis and Selected Associated Vulnerability Factors among Rural Populations in Six Districts of India: A Community-Based Cross-Sectional Study

PONE-D-26-27950R1

Dear Dr. Anand,

We’re pleased to inform you that your manuscript has been judged scientifically suitable for publication and will be formally accepted for publication once it meets all outstanding technical requirements.

Within one week, you’ll receive an e-mail detailing the required amendments. When these have been addressed, you’ll receive a formal acceptance letter and your manuscript will be scheduled for publication.

An invoice will be generated when your article is formally accepted. Please note, if your institution has a publishing partnership with PLOS and your article meets the relevant criteria, all or part of your publication costs will be covered. Please make sure your user information is up-to-date by logging into Editorial Manager at Editorial Manager® and clicking the ‘Update My Information' link at the top of the page. For questions related to billing, please contact billing support.

If your institution or institutions have a press office, please notify them about your upcoming paper to help maximize its impact. If they’ll be preparing press materials, please inform our press team as soon as possible -- no later than 48 hours after receiving the formal acceptance. Your manuscript will remain under strict press embargo until 2 pm Eastern Time on the date of publication. For more information, please contact onepress@plos.org.

Kind regards,

Yasir Alvi

Academic Editor

PLOS One

Additional Editor Comments (optional):

Reviewers' comments:

Reviewer's Responses to Questions

**Comments to the Author**

Reviewer #2: (No Response)

Reviewer #3: All comments have been addressed

**********

2. Is the manuscript technically sound, and do the data support the conclusions?

Reviewer #2: Partly

Reviewer #3: Yes

**********

3. Has the statistical analysis been performed appropriately and rigorously?

Reviewer #2: Yes

Reviewer #3: Yes

**********

4. Have the authors made all data underlying the findings in their manuscript fully available?

The PLOS Data policy

Reviewer #2: Yes

Reviewer #3: Yes

**********

5. Is the manuscript presented in an intelligible fashion and written in standard English?

Reviewer #2: Yes

Reviewer #3: Yes

**********

Reviewer #2: Revisions as suggested previously for table 1( write number of households)and Table 6 needs to be done. Rest all is okay.

Reviewer #3: (No Response)

**********

what does this mean?). If published, this will include your full peer review and any attached files.

If you choose “no”, your identity will remain anonymous but your review may still be made public.

**Do you want your identity to be public for this peer review?**  For information about this choice, including consent withdrawal, please see our Privacy Policy

Reviewer #2: No

Reviewer #3: **Yes:**  Dr. Sameena Ahmad

# Editor report: Acceptance letter

**DOI:** 10.1371/journal.pone.0360758.r004

**Authors:** Yasir Alvi

PONE-D-26-27950R1

PLOS One

Dear Dr. Anand,

I'm pleased to inform you that your manuscript has been deemed suitable for publication in PLOS One. Congratulations! Your manuscript is now being handed over to our production team.

At this stage, our production department will prepare your paper for publication. This includes ensuring the following:

* All references, tables, and figures are properly cited

* All relevant supporting information is included in the manuscript submission,

* There are no issues that prevent the paper from being properly typeset

You will receive further instructions from the production team, including instructions on how to review your proof when it is ready. Please keep in mind that we are working through a large volume of accepted articles, so please give us a few days to review your paper and let you know the next and final steps.

Lastly, if your institution or institutions have a press office, please let them know about your upcoming paper now to help maximize its impact. If they'll be preparing press materials, please inform our press team within the next 48 hours. Your manuscript will remain under strict press embargo until 2 pm Eastern Time on the date of publication. For more information, please contact onepress@plos.org.

You will receive an invoice from PLOS for your publication fee after your manuscript has reached the completed accept phase. If you receive an email requesting payment before acceptance or for any other service, this may be a phishing scheme. Learn how to identify phishing emails and protect your accounts at https://explore.plos.org/phishing.

If we can help with anything else, please email us at customercare@plos.org.

Thank you for submitting your work to PLOS One and supporting open access.

Kind regards,

PLOS One Editorial Office Staff

on behalf of

Dr. Yasir Alvi

Academic Editor

PLOS One
